# Chemical, Biochemical, and Structural Similarities and Differences of Dermatological cAMP Phosphodiesterase-IV Inhibitors

**DOI:** 10.1016/j.jid.2024.10.597

**Published:** 2024-11-27

**Authors:** Jimin Wang, Minh Ho, Christopher G. Bunick

**Affiliations:** 1 Department of Molecular Biophysics and Biochemistry, Yale University, New Haven, Connecticut, USA; 2 Department of Dermatology, Yale School of Medicine, New Haven, Connecticut, USA; 3 Program in Translational Biomedicine, Yale School of Medicine, New Haven, Connecticut, USA

**Keywords:** Atopic dermatitis, PDE4B, PDE4D, Psoriasis skin therapy, Structural biology

## Abstract

Roflumilast, the third phosphodiesterase-IV (PDE4) inhibitor approved for use in dermatology, is indicated for topical treatment of psoriasis, seborrheic dermatitis, and atopic dermatitis, whereas its 2 predecessors, apremilast and crisaborole, are indicated for oral treatment of psoriasis and topical treatment of atopic dermatitis, respectively. All 3 are rationally designed PDE4 inhibitors, but roflumilast is the most potent and effective among the 3, with in vitro inhibitory constant half-maximal inhibitory concentration value of 0.7 nM (roflumilast), 0.14 μM (apremilast), and 0.24 μM (crisaborole), representing differences of over 3 orders of magnitude. PDE4 is a cAMP (an intracellular secondary messenger) hydrolase consisting of at least 4 subtypes of exon-spliced isoforms, which are primarily expressed in immune cells for inflammatory response. PDE4 inhibition lengthens the duration of cAMP signals and increases cellular cAMP concentrations, generating anti-inflammatory effects. We examined the physicochemical principles that make PDE4 inhibitors effective and propose chemical modifications to improve them. Sequence alignment of the catalytic domains of all phosphodiesterases identified many previously unreported invariant residues. These residues bind 1 Zn and 1 Mg ion plus 5 structural water molecules for orienting an attacking μ-hydroxyl/μ-oxo anion and for stabilizing 2 nonbridging phosphate oxygen atoms. The arrangement of the 2 divalent metal ions in phosphodiesterases is not related to that of the classic mechanism for general phosphoryl transfer.

## INTRODUCTION

**Figure 1.**
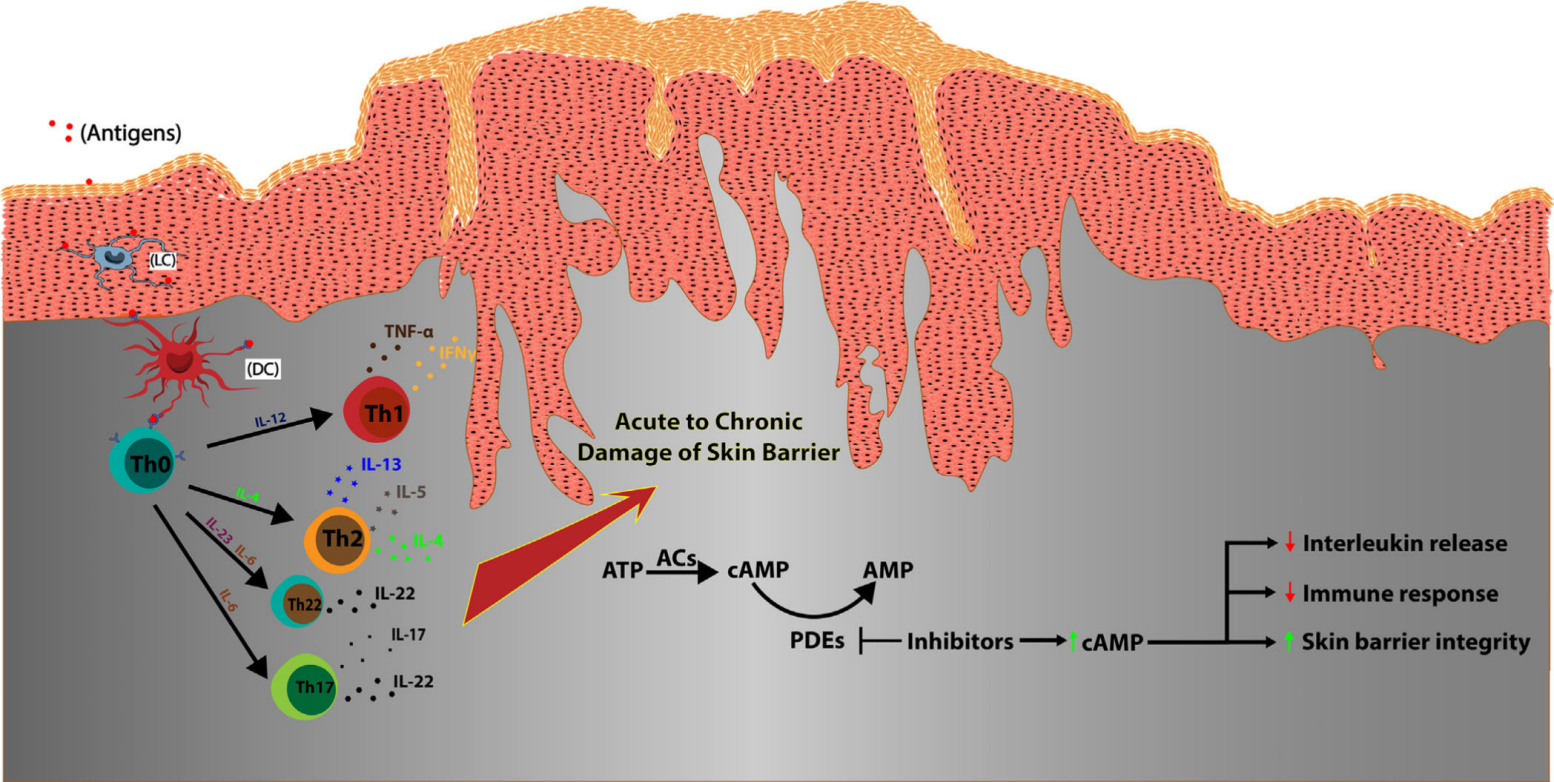
PDE4 enzyme is involved in multiple inflammatory pathways relevant to skin disease. Upon arriving at the skin barrier, antigens activate LCs that reside in the stratified squamous epidermal layer to become mature DCs. Upon maturation, DCs initiate differentiation of naive T cells (Th0) into Th1, Th2, Th17, and Th22. Because type I cytokines (IFNγ, TNF-α), type II ILs (IL-4, IL-5, IL-13), and type III ILs (IL-22, IL-23) function upstream of PDE4 enzyme, this makes PDE4 a central regulator for inflammatory skin diseases such as atopic dermatitis, psoriasis, and seborrheic dermatitis. PDE4 enzyme hydrolyzes cAMP into AMP, and inhibition of this process by PDE4 inhibitors reduces skin inflammation across multiple inflammatory pathways, including Th1, Th2, and Th17 pathways. AC, adenylyl cyclase; AMP, adenosine monophosphate; DC, dendritic cell; LC, Langerhans cell; PDE4, phosphodiesterase-IV; Th, T helper.

cAMP is a universal secondary messenger, synthesized by a family of adenylyl cyclases (ACs) and hydrolyzed by an opposite family of phosphodiesterases (PDEs) to modulate the strength of intracellular signals by controlling the intracellular cAMP concentration (Figure 1) (Hanoune and Defer, 2001; Omori and Kotera, 2007; Ostrom et al, 2022; Sunahara et al, 1996). There are 9 isoforms of membrane-bound ACs and 1 soluble AC form, which are located randomly in multiple chromosomes (Gaudin et al, 1994). Membrane-bound ACs are integrators of transmembrane signal transduction through G-protein coupled receptors, which are divided into stimulatory (G*_s_*) or inhibitory (G*_i_*) classes for controlling the ACs’ rate of cAMP synthesis (Ostrom et al, 2022; Yang et al, 2021). There are 11 classes of PDEs encoded by 21 genes, located in multiple chromosomes (Conti and Beavo, 2007; Omori and Kotera, 2007). Each class has several isoforms resulting from different exon-spliced products for hydrolysis of cAMP, cGMP, or both. Dysregulation of cAMP concentrations associated with overactive ACs or overactive PDEs causes many human diseases, such as stroke (PDE4D), retinal degeneration (PDE6), endocrine disorders (PDE11A), and CNS disorders (PDE4B) (Conti and Beavo, 2007; Keravis and Lugnier, 2012; Zuo et al, 2019). Therefore, rationally designed inhibitors targeting either ACs or PDEs were developed for clinical use (Li et al, 2018; Paes et al, 2021; Seifert et al, 2012). Some designed drugs are bifunctional, simultaneously targeting both G-protein coupled receptors and PDEs (ie, GS-5759) (Joshi et al, 2017). In fact, G-protein coupled receptors are the single largest protein family targeted by Food and Drug Administration (FDA)–approved drugs (Hauser et al, 2017; Insel et al, 2019; Sriram and Insel, 2018).

Soluble cAMP-specific PDE4 has 4 isoforms (PDE4A–D) and is highly expressed in keratinocytes and immunocytes (including T cells, monocytes, macrophages, neutrophils, dendritic cells, eosinophils), which modulate inflammation (Figure 1) (Chiricozzi et al, 2016; Li et al, 2018). It is also highly expressed in smooth muscles, in epithelial cells for maintaining epithelial integrity, and in brain cells where neuroinflammation and other neuron injuries occur (Chiricozzi et al, 2016; Li et al, 2018). PDE4 isoforms provide immunological balance and homeostatic regulation of intracellular cAMP, which controls the state of many phosphoproteins (also called phosphoproteomes) in the cells through the cAMP-dependent protein kinase A (PKA) pathway. It also stimulates other immune responses through cAMP-generating signals and agents (Epstein, 2017). PKA regulates gene expression of various PDE4 isoforms under different promoters as well as the enzymatic activation of certain PDE4 isoforms (MacKenzie et al, 2002; Manganiello, 2002).

A long isoform of PDE4 contains a single Ser residue (S133) in UCR1 and remains autoinhibited, before its phosphorylation, by a block in its catalytic site in trans through dimerization involving both UCR1 and UCR2 (Cedervall et al, 2015; Xie et al, 2014). It is activated once Ser133 is phosphorylated by PKA, establishing a negative feedback loop (MacKenzie et al, 2002). However, phosphorylation of another Ser residue (S659, immediately outside the crystallographically defined C-terminal end of the catalytic domain) of PDE4D3 by the extracellular receptor stimulated kinase extracellular signal–regulated kinase 2 (p42^MAPK^) significantly reduced PDE4 activity (by ~75%), which can be restored by protein phosphatase 1 (Hoffmann et al, 1999), highlighting complex cross-talk between PKA and extracellular signal–regulated kinase 2–MAPK signaling pathways. Different classes and isoforms of PDEs form a variety of large protein complexes that regulate the amplitude and duration of cAMP signals (Aslam and Ladilov, 2022; Conti and Beavo, 2007; Zaccolo et al, 2021).

Another long isoform of PDE4D contains a phosphorylation-dependent protein degradation (phosphodegron) motif that is subject to dual phosphorylation by casein kinase 1 and GSK3β for phosphorylation-dependent protein degradation through the ubiquitin-proteasome system, whereas it is protected by protein phosphatase 2B (calcineurin) through dephosphorylation (Zhu et al, 2010). When different classes and isoforms of PDEs are present in autoinhibited states in the cell, they rapidly respond to high-amplitude and short-duration excessive cAMP signals. They are often subject to proteolysis through the ubiquitin-proteasome system and short lived. Some PDEs are long lived for dealing with constitutive hydrolysis of cAMP signals and transcribed through cAMP–CREB proteins (Carlezon et al, 2005; Shaywitz and Greenberg, 1999). These long-lived PDEs belong to short forms of PDE4 isoforms with half-life of 1–2 hours and are difficult to turn off. This makes PDE4-specific inhibitors ideal drugs for maintaining a basal cAMP level.

Inhibition of PDE4 by externally supplied inhibitors is predicted to upregulate the PKA signaling pathway by increasing cellular cAMP concentration, which is closely associated with the suppression of overactive immune responses and their intermediates (Figure 1) (Li et al, 2018). Elevated cellular cAMP level upon PDE4 inhibition increases the function of T cells while exhibiting little effect on B cells (Sakkas et al, 2017; Schafer et al, 2014). Treatment with cAMP-specific PDE4 inhibitor rolipram enhances cAMP PKA-dependent proteasome activity and reduces protein misfolding diseases such as taupathy (Myeku et al, 2016). The elevated cAMP level in keratinocytes and epithelial cells can also inhibit inflammatory responses and regulate cellular barrier function and cell growth (Pagès et al, 2009).

**Figure 2.**
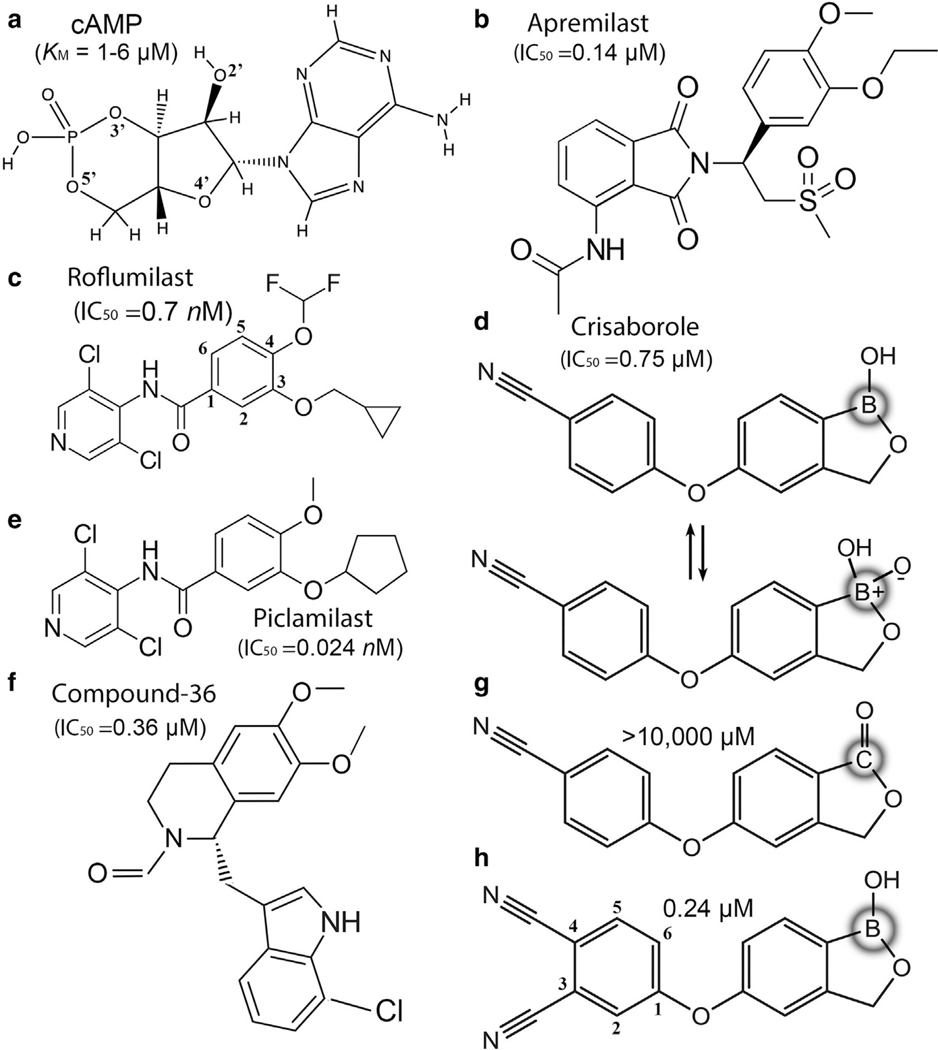
Chemical structures of the cAMP substrate and 3 FDA-approved inhibitors of PDE4 and their derivatives with reported kinetic and inhibition properties. (**a**) The cAMP substrate. (**b**) Apremilast or N-{2-[(1S)-1-(3-ethoxy-4-methoxyphenyl-2-(methylsulfonyl)ethyl]-1,3-dioxy-2,3-dihydro-1H-isoindoyl-4-yl} acetamide. (**c**) Roflumilast or N-(3,5-dicholorpyridin-4-yl)-3-cyclopropylmethoxy-4-difluoromethyoxy-benzamide. (**d**) Crisaborole or 5-(4-cyanophenoxy)-1,3-dihyro-1-hydroxy-[2,1] benoxaborole and its zwitterionic form after hydration. (**e**) Piclamilast, a derivative to roflumilast. (**f**) Compound-36, a derivative of apremilast after extensive modifications, whose complex structure is discussed in the text. (**g**) Replacement of boron in crisaborole with carbon decreases inhibition capacity by over 10,000-fold. (**h**) An insertion of an extra cyano group at 3 position of pyrin ring improves inhibition capacity by 3-fold, whose complex structure is also discussed in the text. FDA, Food and Drug Administration; IC_50_, half-maximal inhibitory concentration; PDE4, phosphodiesterase-IV.

Roflumilast, recently FDA approved for topical psoriasis, seborrheic dermatitis, and atopic dermatitis treatment, was initially approved by the European Union in 2010 and the United States in 2011 to reduce chronic obstructive pulmonary disease exacerbations in patients with severe chronic obstructive pulmonary disease associated with chronic bronchitis (Figure 2) (Boswell-Smith and Spina, 2007; Li et al, 2018). Apremilast was FDA approved in 2014 for adult patients with active psoriatic arthritis and moderate-to-severe plaque psoriasis, and crisaborole was FDA approved in 2016 for topical treatment of mild-to-moderate atopic dermatitis in patients aged ≥2 years (Li et al, 2018). The reported PDE4 half-maximal inhibitory concentration is 0.14 μM for apremilast, 0.24 μM for crisaborole, and 0.7 nM for roflumilast (Table 1 and Figure 2) (Bender and Beavo, 2006; Card et al, 2004; Fabbri et al, 2010; Freund et al, 2012; Zhang et al, 2021, 2013, 2004). Moreover, apremilast was or is in numerous clinical trials for other inflammatory diseases (Li et al, 2018). In this study, we evaluate the structural properties of these inhibitors and related compounds and describe physicochemical and mechanistic insights into how they effectively inhibit PDE4. We also provide insights into a large number of invariant residues important to the catalytic function of PDEs and discuss PDE4 inhibitor designs.

Whereas large public databases of PDE4 inhibitors exist (Card et al, 2004; Jansen et al, 2016; Li et al, 2018; Zhang et al, 2021, 2013), pharmaceutical companies may contain more structural and biochemical information about PDE4-–inhibitor complexes than publicly available. Nevertheless, given sufficiently large biochemical and structural databases available, machine learning and artificial intelligence can often generate reliably any missing structural or biochemical information (Niu et al, 2013), which will foster the next generation of PDE4 inhibitor design. An effective PDE4 inhibitor exhibits high enzyme specificity so that it avoids unintended off-target effects, minimizes side effects, and utilizes the smallest amount of active ingredient. Roflumilast’s inhibition constant for PDE4 is around 0.7 nM, but it is >200 μM (300,000-fold selectivity) for 7 of the remaining 9 human PDEs measured (Table 1). Its derivative piclamilast (0.024 nM) further increases the inhibition constant by over an order of magnitude against PDE4, but this comes with a loss in specificity for PDE4 because it also increases inhibition to many other human PDEs (Table 1). Piclamilast differs from roflumilast in 2 substituents: 4-difluoromethoxy was replaced with methoxy (ie, removal of 2 fluoride atoms), and 3-cyclopropanylmethoxy was replaced with cyclopentylmethoxy (ie, making it larger and more hydrophobic) (Figure 2). The 2 same substitutions of piclamilast at the 3,4-positions are also present in rolipram, whose cocrystal structure with PDE4B revealed the same binding mode as piclamilast (Card et al, 2004; Cedervall et al, 2015). Rolipram and piclamilast (RP73401) are among the first generation of PDE4 inhibitors, patented for their synthesis in 1993 (Ashton et al, 1994; Beeley and Millican, 1993), whereas roflumilast was patented for its synthesis in 1995 (Flockerzi et al, 1995). Roflumilast can completely block the PDE4 hydrolysis of cAMP because the reported Michaelis–Menten constant *K*_M_ of PDE4 for cAMP is only ~1–6 μM (Table 1).

AC catalyzes the cyclization reaction of adenosine triphosphate, starting with deprotonation of 3’-OH as a nucleophile, ending with the cAMP product plus the leaving product pyrophosphate, which is further hydrolyzed to inorganic phosphates by pyrophosphatase, making the reaction irreversible. PDE catalyzes cAMP hydrolysis, starting likely with a hydroxyl nucleophile, and ending with the AMP containing a protonated 3’-OH through an undefined proton transfer event associated with the reaction (Huai et al, 2003; Liu et al, 2008; Wang et al, 2007a, 2007b). However, the proposed hydrolysis mechanism has unresolved issues associated with disputed interpretation of Asp-to-Asn mutant structures, as discussed below. The nucleophile is an μ-hydroxyl or μ-oxo anion bridged by 2 divalent metal ions, which is distinct from the classic 2-metal-ion catalytic mechanism proposed by Steitz for general phosphoryl transfer reactions (Beese and Steitz, 1991; Steitz, 1999; Steitz and Steitz, 1993). HIV integrase uses the classic 2-metal-ion mechanism for integration of viral sequences into the human genome through strand exchange reaction (Dyda et al, 1994). The 2 metal ions bound in the integrase active site were targeted for designing inhibitors with nM binding affinity (Goldgur et al, 1999; Smith et al, 2021). This inhibitor design concept should be applicable to the 2 metal ions of PDE4. Striking resemblance exists between known G-protein coupled receptor–PDE4 bifunctional inhibitors (ie, GS-5759) and HIV integrase inhibitors (Joshi et al, 2017), opening the possibility of a new functional role for targeting the divalent metal ions bound in the catalytic site of PDE4.

This study provides an objective and independent analysis on published PDE4 research; on its crystallographic complexes with inhibitors, substrates, and products; on its catalytic mechanism; and on mechanistic and structure-based drug design. All figures prepared for this manuscript are original, including multiple sequence alignment, structural comparisons, and corrections for misinterpretations of some published crystallographic and mutational data.

## RESULTS AND DISCUSSION

### Structural and sequence conservation of PDEs

**Figure 3.**
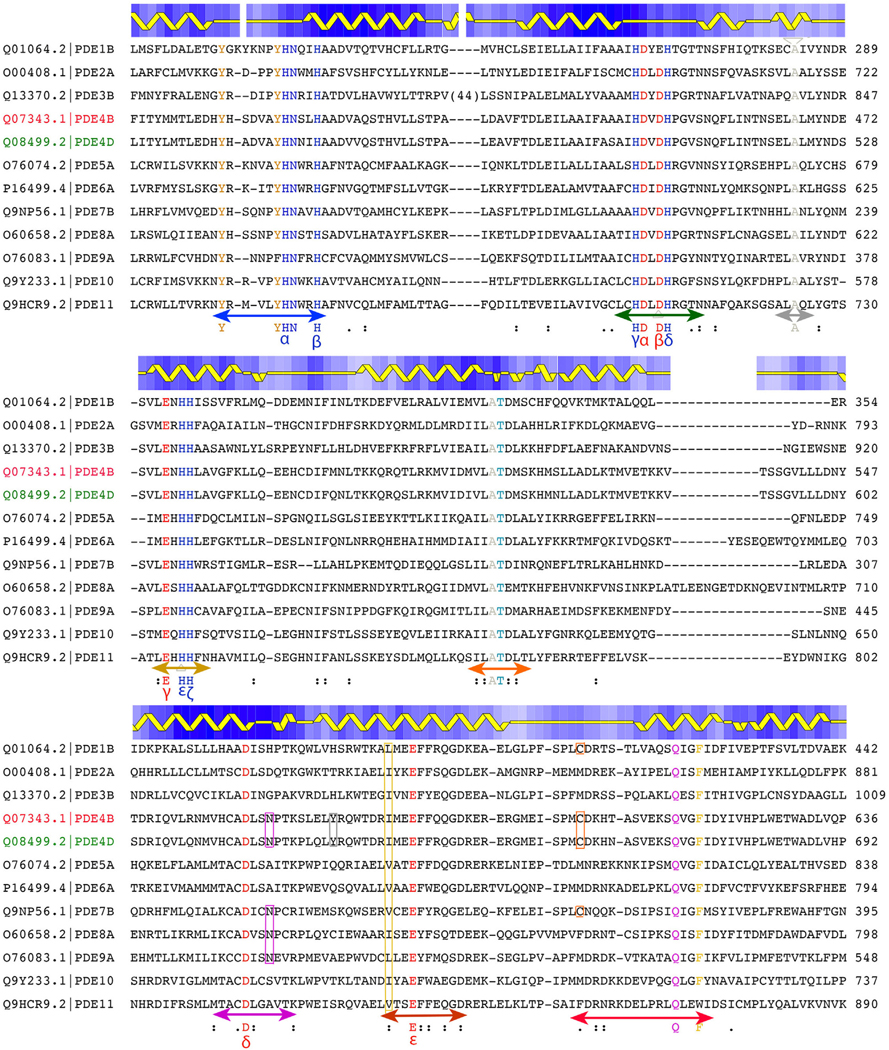
Multiple sequence alignment of the catalytic domain of all human PDEs using ClustalW. Invariant and almost invariant (see text for definition) residues are colored, which form both the catalytic site and cAMP-binding site with 3 exceptions in gray triangles. Six invariant His residues are labeled using Greek alphabet (α, β, γ, δ, ε, and ζ), and so are 5 invariant carboxylate residues (α, β, γ, δ, and ε). Three exceptions are single invariant Ala residue (gray down triangle, in the third sequence motif away from the catalytic site) and 1 Asp (β) and His (ε) pair (gray up triangles). The single invariant Gln residue in magenta recognizes N1 (and in some structures also N6) of cAMP through side chain HBs. Hydrophobic residues Ile in yellow box in the seventh sequence motif and Phe in yellow in the QVGF motif form a clamp, flanking both sides of the cAMP adenine ring. Magenta (Asn residues in the sixth sequence motif), orange (Cys residues in the eighth sequence motif), and gray (Y329) -boxed residues are PDE4-specific residues (details are provided in the text). Eight conserved sequence motifs are A (blue), B (forest green), C (gray), D (brown), E (light salmon), F (magenta), G (dark salmon), and H (red). Secondary structures were prepared using the program Procheck using the 1ptw/PDE4D2 coordinates. Additional 63 residues at the N-terminus and 22 residues at the C-terminus of the catalytic domain were omitted for clarity in this plot because they do not contain any conserved motifs and are away from the catalytic site. HB, hydrogen bond; PDE, phosphodiesterase.

The primary sequence for the C-terminal catalytic domain of 11 classes of human PDEs is highly conserved according to alignment using ClustalW; however, there are large variations in N-terminal sequences, in their lengths, and in some conservations beyond this study’s scope (Figure 3) (Thompson et al, 1994). This alignment differs from an earlier one used for identification of the so-called HD metal ion-dependent catalytic superfamily with a motif HX_3_HX_25–35_(D/E) across different phosphohydrolases from different species (Aravind and Koonin, 1998). The secondary structure assignment for the aligned sequences was made using Procheck for the 1ptw (Protein Data Bank identification)/PDE4D2 coordinates (Huai et al, 2003; Laskowski et al, 1993). Among 12 sequences (1 from each of 10 classes plus 2 subclasses from PDE4, ie, PDE4B and PDE4D) of the catalytic domain, 15 residues are invariant, distributed in 8 conserved sequence motifs A to H, including 6 His residues (named using Greek alphabet, α, β, γ, δ, ε, and ζ, according to their order of appearance in sequence), 2 Asp, 2 Glu, 2 Ala, 1 Thr, 1 Tyr, and 1 Gln residues (Figure 3). Almost invariant residues (defined by 11 identical sequences plus 1 sequence of similar hydrophobicity) include 1 Tyr (11 Tyr plus 1 Phe), 1 Asp (11 Asp plus 1 Glu), and 1 Phe (11 Phe plus 1 Trp) residues. Five conserved carboxylates are also named using Greek alphabet (α, β, γ, δ, and ε).

An advantage of using the Greek alphabet for naming conserved residues is to avoid conflicting/confusing sequence numbers of different PDE isoforms or even different numbers among the same isoform by various investigators. For example, the 1tpw crystal structure cited the Q08499.2 Uniport sequence (PDE4D2) for its subclone, but the residue numbering of the 1tpw structure differs from the Uniport sequence by 309, which may reflect different isoforms within a subclass used for crystallographic analysis for unknown historical reasons. We label residue numbers in this paper according to what the corresponding coordinates provide (but not the long isoforms of the unified Uniport numbers).

**Figure 4.**
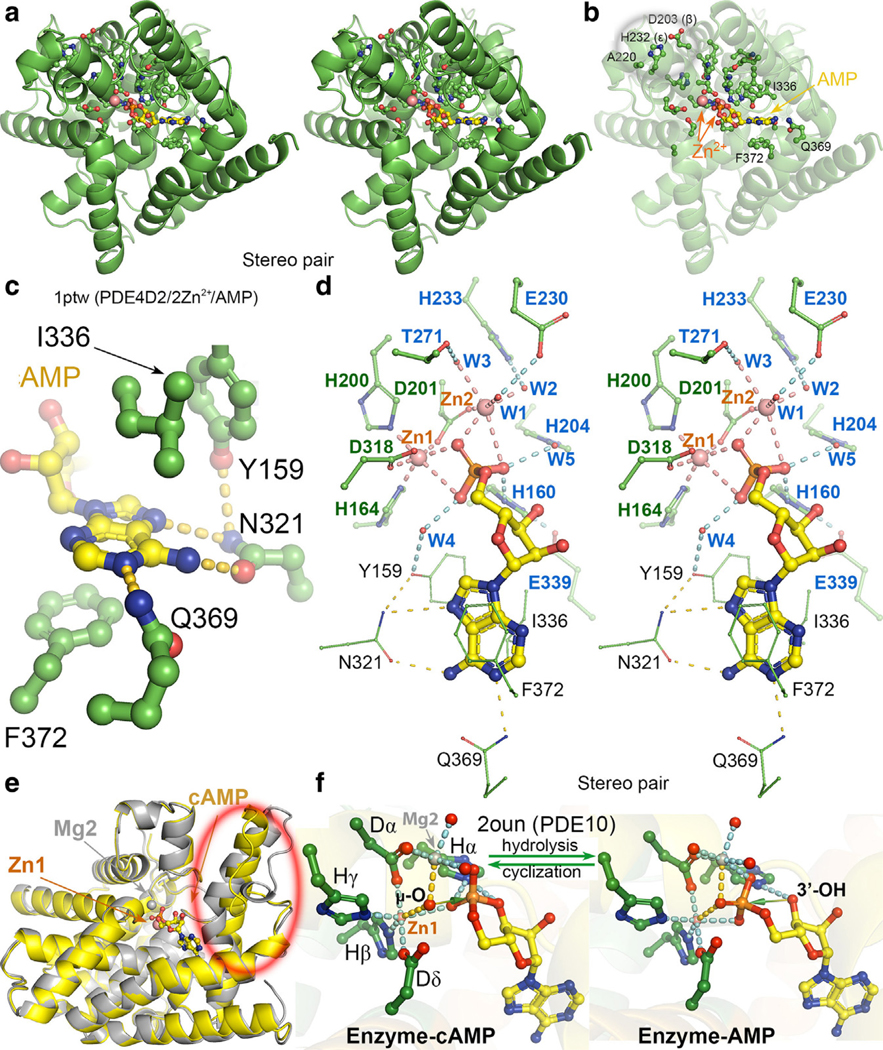
Catalytic mechanism of cAMP PDE4 hydrolase. (**a**) Stereodiagram of all conserved residues among PDEs in the 1ptw structure from which the initial proposed mechanism was based on the enzyme–AMP product complex with 2 Zn^2+^ ions, Zn1 and Zn2. (**b**) The same view as **a** with cartoons faded away and 3 conserved residues not associated with the catalytic site circled in gray at top left. (**c**) Recognition of the cAMP adenine base by Q369 and N321 with 2 residues F372 and I366 (an arrow indicates its Cα) flanking both sides of the cAMP adenine ring. (**d**) Stereodiagram of the conserved residues in the following 3 classes: (i) thin residues for recognition of the base (as in **c**), (ii) residues in green for binding of 2 metal ions, and (iii) residues in blue for binding of 5 internal water molecules that are either ligands to the second metal ion or hydrogen bonded to 2 phosphate oxygen ligands (Supplementary Movie S1 for this panel). (**e**) Superposition of 2 monomers of cGMP PDE10 in the crystallographic asymmetric unit of the 2oun structure, one (yellow) with cAMP inhibitor bound and the other (gray) without it. Red circle shows different conformations between the 2 monomers. (**f**) Corresponding catalytic mechanisms: (i) the bridging μ-oxo anion between Mg^2+^ and Zn^2+^ ions is a nucleophile for attacking the cyclic phosphate center during hydrolysis; Hα (H525) is a proton donor for stabilizing the leaving O3’ group, and (ii) Hα deprotonates O3’ to generate an O3’ anion as a nucleophile for the reverse cyclization reaction. AMP, adenosine monophosphate; PDE, phosphodiesterase.

With exception of 1 invariant Ala and 1 invariant His plus 1 almost invariant Asp, the remaining 15 residues are all distributed immediately surrounding the cAMP/cGMP-binding pocket (Figure 4). They are involved directly in recognition of the cAMP substrate and in binding of 2 catalytic metal ions and their ligands or indirectly play important roles for maintaining conformations of other invariant residues. The 3 exceptions are single invariant Ala residue in motif C and 1 hydrogen bonded Asp (β)-His (ε) pair in motifs B and D, respectively, all of which are near the dimeric interface of some truncated PDE4B and PDE4D catalytic domains and not directly involved in catalysis or substrate/product binding. They could be involved in regulation and dimerization of PDEs, in interactions with other proteins in large regulatory complexes, and in pH-dependent regulation of PDE’s functions.

The 2 catalytic metal ions of PDEs are 1 Zn^2+^ ion (Zn1) and 1 Mg^2+^ ion (Mg2). When Mg^2+^ is not included in crystallization, Zn^2+^ can also occupy the Mg2 site. The Zn1-binding site is formed by His (β) (motif A), His (γ) and Asp (α) in motif B, and Asp (δ) in motif F; the Mg2 site is formed by both Asp (α) and Asp (δ) (Figure 3). In both apo and enzyme–cAMP substrate complex structures, Zn1 and Mg2 share a μ-bridging hydroxyl anion (Figure 4f). In the crisaborole complex structure (discussed below), the phosphate mimic binds the 2 divalent metal ions with its 3 oxygen atoms.

The adenine of cAMP is recognized at N1 by Q369 in PDE4B and PDE4D (motif H) (Figure 4c), an invariant residue among all PDEs of either cAMP or cGMP in substrate specificity. Q369 sidechain orientation is maintained by Y329, present only in PDE4B and PDE4D (see below drug design for stereodiagram) but not in other PDEs (Figure 3). When Y329 is absent in other cAMP PDEs, the Q369 sidechain carbonyl is also H bonded to N6 of cAMP. Both N6 and N7 of cAMP are also H bonded to the N321 sidechain in motif F in PDE4s. This Asn residue is present at the same position in all cAMP-specific PDE4B, PDE4D, PDE7B, and PDE8A that generally do not bind cGMP (Figure 3). The orientation of N321 is maintained by its H bonding to Y159, which is an invariant residue of all PDEs in motif A. Interestingly, an Asn residue in motif F is also present in PDE9A, which prefers cGMP over cAMP as a substrate. Both sides of the adenine base ring are flanked by F372, which is almost invariant in motif H, and by I336, which is always a hydrophobic residue at this position in motif G among all PDEs (Figure 3 and Figure 4c).

When PDEs use cGMP as a substrate, they often exhibit poor substrate selectivity between cGMP and cAMP with the exception of 3 PDEs—PDE5A, PDE6A, and PDE9A—which prefer cGMP over cAMP as a substrate by 50- to 220-fold (Table 1). This substrate specificity is defined by the orientation of the invariant Gln residue in motif H. When this sidechain is fixed in one orientation, it recognizes both N1 and N6 of only cAMP, but when it is reoriented in the flipped orientation, it recognizes N1 and O6 of only cGMP (Zhang et al, 2004), that is, it binds both cAMP and cGMP, known as a glutamine switch (Wang et al, 2007b; Zhang et al, 2004). The cGMP-specific PDEs often rely on other regulatory domains to enhance substrate selectivity such as GAF (cGMP-activated PDEs, ACs, and Fh1A) domain (Conti and Beavo, 2007; Martinez et al, 2002a, 2002b). Some PDEs such as PDE2A are dual-specific enzymes that break down both cAMP and cGMP cyclic nucleotides (Calamera et al, 2022; Cardarelli et al, 2024; Zaccolo and Movsesian, 2007). They differ from many other adenine-specific or guanine-specific proteins (or enzymes) where the electrostatic potential properties of proteins alone can sufficiently define their nucleotide selectivity in addition to specific hydrogen donor/acceptor arrangements (Basu et al, 2004; Issa et al, 2022).

In the adenosine monophosphate (AMP)–bound complex of PDE4B and PDE4D, the attacking μ-hydroxyl anion or μ-oxo anion—bridged between 2 divalent metal ions—is replaced with the oxygen atom of the product phosphate, which differs from the classic 2-metal-ion mechanism for the phosphoryl transfer reaction where the attacking nucleotide interacts with only 1 of the 2 divalent metal ions. Two non-bridging oxygen of the AMP phosphate become 2 ligands: one for each of the 2 metal ions in both the enzyme–substrate complex and the enzyme–AMP product complex (Figure 4d and Supplementary Movie S1) (Huai et al, 2003). In the apo structure and some inhibitor-bound complexes, these 2 positions are occupied by 2 water molecule ligands. Mg2 has 3 additional water molecule ligands W1, W2, and W3 that are indirectly involved in catalysis (Figure 4d). These 3 ligands are rigidly in place through hydrogen bonds (HBs)—one each by 3 conserved residues—to orient the attacking μ-hydroxyl anion: E230 (in the 1ptw coordinates or Eγ), H233 (Hζ) in motif D, and T271 in motif E (Huai et al, 2003). Two nonbridging phosphate oxygen atoms of both cAMP and AMP, which become the planar triangle base in the pentavalent transition state, are further bound to 2 water molecules W4 and W5, which are fixed by the invariant H204 residue (Hδ) in motif B and Y159 residue in motif A, respectively (Figure 4d). Therefore, 5 ordered waters W1–W5 function as extension arms of the invariant residues and form an integral part of the PDE enzymes in both the enzyme–substrate and enzyme–product (EP) complexes.

### Catalytic mechanism of PDEs

In the 2 Zn/AMP bound EP complex, H160 (Ha) in motif A is H bonded to 1 nonbridging phosphate oxygen atom and to E339 (Eε) in motif G (Card et al, 2004). In the Zn1/Mg2-cAMP bound cGMP-specific PDE10 complex (in which cAMP becomes a competitive inhibitor), this His residue also interacts with 3’-O so that it protonates the leaving 3’-hydroxyl group during hydrolysis and deprotonates 3’-hydroxyl during the reverse cyclization reaction (Figure 4f) (Wang et al, 2007a). The Glu–His pairing makes the His residue a good base for deprotonation of 3’-OH for the cyclization reaction but a poor acid for protonation of the 3’-O group during the hydrolysis, which requires the PDE10 enzyme to take a proton somewhere, perhaps from the attacking nucleophile through a proton tunneling mechanism. The understanding of the protonation state of the nucleophile remains incomplete. In the apo form, the bridging ligand is likely to be a hydroxyl anion to have charge neutrality (2 divalent metal ions, Dα, Eγ, and Dδ). However, in the transition-state complex, the proton on the nucleophile hydroxyl anion is likely transferred to the leaving 3’-hydroxyl group, either in sequential or in concomitant manner. When it is in sequential manner, the attacking nucleophile is a μ-oxo anion.

The PDE catalytic mechanism was proposed initially on the basis of a Zn^2+^ ion/AMP EP complex, which has distortions to the Hα residue so that its role could not be definitively assigned (Huai et al, 2003). This issue was fully resolved in other high-resolution crystal structures with Zn1/Mg2-bound complexes and in Asp mutants (Liu et al, 2008; Wang et al, 2007b). However, interpretations of some DαN-mutant complex structures were problematic as discussed below, which could prevent full elucidation of the complete catalytic mechanism (both Dα and Dδ reside in motif B). They should be excluded in mechanistic consideration. A similar catalytic mechanism was proposed on the basis of the PDE9 structures of both enzyme–substrate, EP, and E+P (or EP’) complexes in which 1 catalytic divalent metal ion was replaced with Na^+^ ion (Liu et al, 2008; Wang et al, 2007b).

For the D674A (ie, DδA) mutant of PDE10A2, there is only 1 Mg2 ion bound in both the apo (2ous) and cAMP-inhibited (2our) structures (each 1.45 Å resolution), and Zn1 ion was missing, as expected (Wang et al, 2007a). However, for the D564N (DαN) mutant of PDE10A2 in both the apo (2ouv at 1.56 Å resolution) and cAMP-inhibited (2ouy at 1.9 Å resolution) complexes, both Zn1 and Mg2 sites were unexpectedly occupied by respective divalent metal ions (Wang et al, 2007a). For the D564N sidechain amide to be a ligand for 2 divalent ions, it must be deprotonated to become an anion, which disagrees with known Asn chemical properties. Because DαN mutant cannot simultaneously bind 2 divalent metal ions, the observation of the apparent 2 divalent metal ions in this mutant implies that (i) the DαN was post-translationally modified to revert back to an Asp residue to some extent; (ii) the DαN sidechain had 2 alternate conformations through its sidechain flipping, and each bound 1 divalent metal ion, but a mixture of these 2 populations gave the appearance of 2 divalent metal ions; and/or (iii) the identity of metal ions was incorrectly assigned crystallographically.

Examination of the deposited σ_A_-weighted F_o_eF_c_ residual maps for the 2ouv structure at 1.56 Å resolution showed that the Zn1 site had −23.1σ and −15.4σ features for 2 monomers, and the Mg2 site had about −7.0σ features (Wang et al, 2007a), meaning that the number of electrons in these sites were significantly overestimated in the atomic models. Similarly, the deposited s_A_-weighted F_o_eF_c_ residual maps for the cAMP-inhibited 2ouy complex at 1.90 Å resolution also revealed large negative features (−18.5 Å_2_ and −16.8 Å_2_) at the Zn1 site. Moreover, reported B-factor for the Zn^2+^ ion was 34.8 Å_2_ and 22.7 Å_2_ for the 2 molecules of the 2ouv structure, respectively, and that of the Mg^2+^ ion was 34.1 Å_2_ and 24.5 Å_2_ (Wang et al, 2007a), all of which were much higher than those of the corresponding ligands, meaning that the number of electrons was overestimated at these sites, but the increased B-factors were compensating for this error. These findings raise concerns about using these structures for mechanistic considerations of PDE hydrolysis.

The catalytic efficiency of PDEs is further modulated in short-term regulations through dimerization and protein phosphorylation in upstream conserved regions and perhaps other associated proteins (Table 1) (Cedervall et al, 2015; Hoffmann et al, 1999; MacKenzie et al, 2002; Manganiello, 2002; Xie et al, 2014). Without large variable N-terminal domains and sequences, catalytic domains of the PDEs in 2 monomers of a dimer often appear to function independently. However, 2 monomers of the PDE10A2 dimeric complex present in the crystallographic asymmetric unit behaved differently in the presence of 20 mM AMP in crystal soaking solutions (Wang et al, 2007a). One monomer binds AMP and forms an inhibited complex, but the second monomer undergoes many conformational changes to make the nucleotide-binding pocket inaccessible (Figure 4e) (Wang et al, 2007a), highlighting possible negative intersubunit regulation of the 2 monomers within PDE dimers.

An enzyme simply lowers the energetic barrier of the reaction it catalyzes but does not alter its equilibrium (Kraut, 1988). At the transition state, PDEs catalyze both the forward hydrolysis reaction and the reverse cyclization reaction (Figure 4f). What makes hydrolysis catalyzed by PDEs irreversible is the subsequent conformational changes of the enzymes, particularly to move the freed 3’-OH away from the phosphate site. It remains unclear how much product inhibition of PDE4 plays a role in cAMP hydrolysis, which has not been studied extensively. Both cAMP and AMP are master regulators of cellular metabolism through cAMP-dependent PKA and AMP-activated protein kinase pathways, respectively (Hardie, 2011; Russell and Hardie, 2020).

### Structures of roflumilast, piclamilast, and apremilast/PDE4 complexes and their derivatives

Roflumilast, piclamilast, and apremilast share 2 HB acceptors at 2 alkyloxy groups at positions 3 and 4 of the core phenyl ring of benzamide (Figure 2), which are recognized by the sidechain amide of the invariant Q (Q369 in PDE4B and PDE4D) in motif H. Roflumilast and piclamilast differ only in alkyl identities of the 2 alkyloxy groups: roflumilast has 3-cyclopropylmethoxy-4-difluoromethyoxy groups, whereas piclamilast has 3-cyclopentyloxy-4-methoxy groups (Figure 2). Apremilast has an isoindoyl-4-yl acetamide with 2-methylsulfonylethyl group at 2-position connected to the substituted phenyl ring with 3-methoxy-4-ethyoxy substitution groups (Figure 2).

**Figure 5.**
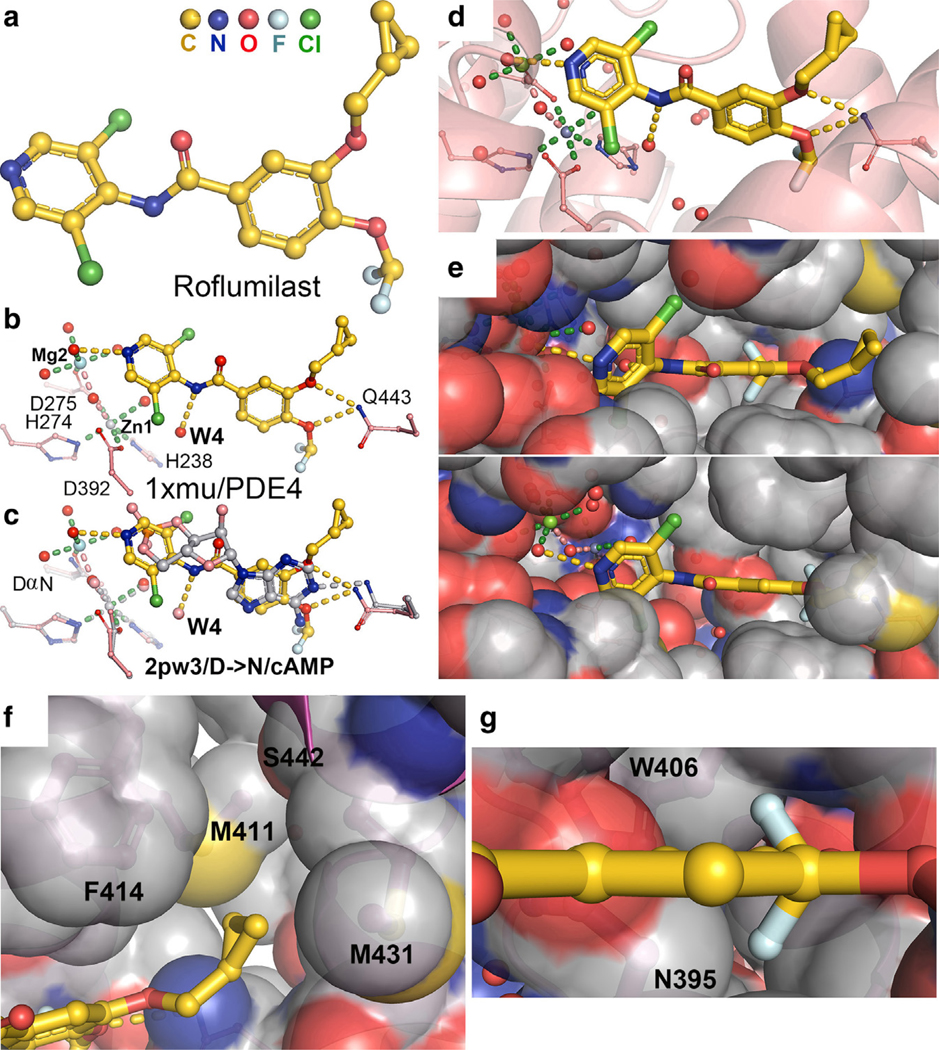
Roflumilast as a competitive inhibitor for PDE4 cAMP hydrolase. (**a**) Chemical structure of roflumilast. (**b**) Recognition of the inhibitor through conserved HBs, including conserved water molecules in the inhibitor-bound complex of PDE4 (1xmu). (**c**) Superposition of the roflumilast–PDE4 complex (1xmu) with the cAMP-bound enzyme–substrate complex (2pw3, silver) with the catalytic D to N mutant. (**d**) The binding environment of roflumilast within the 1xmu complex. (**e**) Two views of surface representation of the PDE4 enzyme. (**f**) A close-up view of the binding pocket for the cyclopropyl methyloxy group. (**g**) A close-up view of the binding pocket for the difluoromethyloxy group. HB, hydrogen bond; PDE4, phosphodiesterase-IV.

Comparison of PDE4D/cAMP complex with PDE4B/roflumilast complex of different isoforms shows that roflumilast is an ideal analog of cAMP (Figure 5) (Card et al, 2004; Wang et al, 2007b). Both 3-methyloxy and 4-methyloxy of the benzamide core of roflumilast form 2 HBs with the invariant glutamine sidechain in the place of N1 of cAMP (Figure 5b–d). The 1-amine of pyridin of roflumilast occupies a position only 1.6 Å away from the cyclic phosphate group of cAMP and forms 1 HB with a Zn-bound water molecule. This pyridin can be modified to simultaneously bind 2 metal ions with 3 HBs using the same procedures as designed inhibitors that are targeting 2 metal ions of HIV integrase. The 4-amine of the pyridin forms 1 HB with a conserved water molecule W4, which can be modified to make W4 be a part of the inhibitor (Figure 5b and c). The presence of the difluoromethyloxy group in roflumilast forces the N395 sidechain to adopt an alternative rotamer from the one in the cAMP-bound complex that recognizes both N7 and N6 of the cAMP adenine base. Otherwise, 1 fluoride atom will clash into the N395 sidechain (Figure 5g). Therefore, roflumilast has similar functional groups for formation of HBs with the enzyme as cAMP and the benzamide core for stacking as the base of cAMP, making it a nearly ideal cAMP analog.

**Figure 6.**
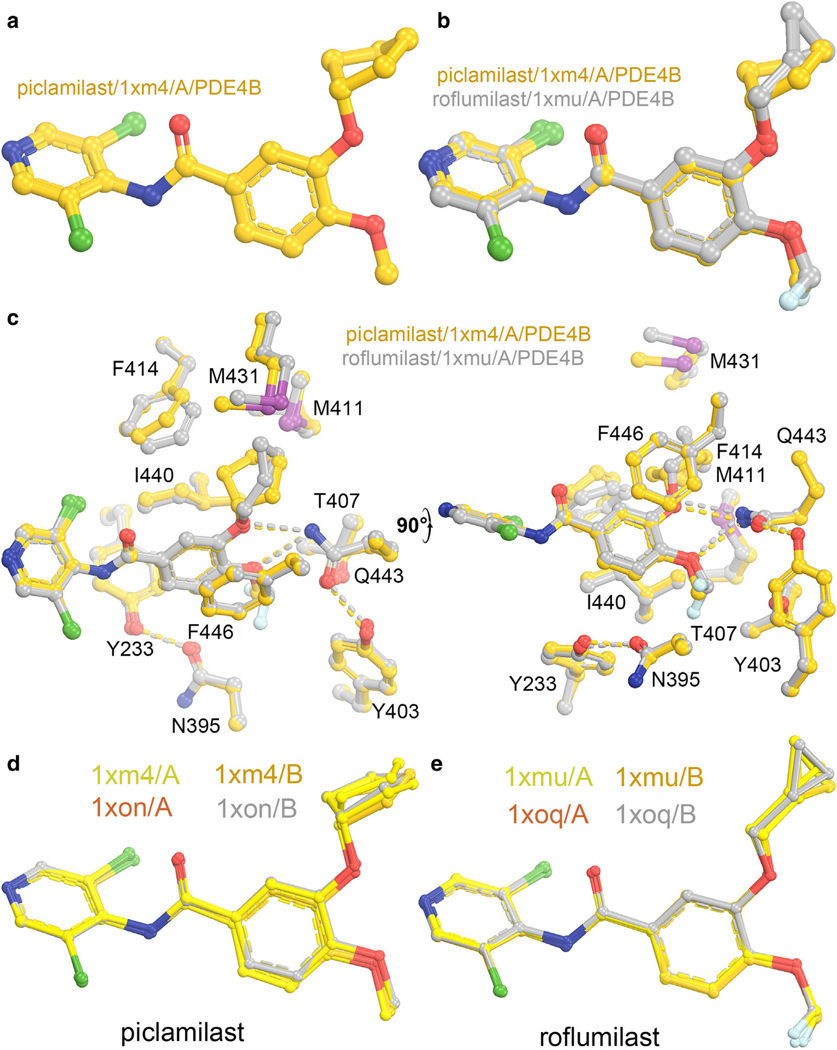
Structural comparison of piclamilast and roflumilast complexes of PDE4B and PDE4D. (**a**) Conformation of piclamilast in the monomer A of the 1xm4 structure of PDE4B. (**b**) Comparison of piclamilast (gold) and roflumilast (silver) complexes of PDE4B (monomer A of 1xm4 and 1xmu structures). (**c**) Two orthogonal views of **b** with sidechains surrounding the different substituents of the 2 compounds. (**d**) Conformations of four copies of piclamilast in 2 monomers of PDE4B and PDE4D structures. (**e**) Conformation of 4 copies of roflumilast in 2 monomers of PDE4B and PDE4D structures. PDE, phosphodiesterase.

The 3-cyclopropylmethoxy group of roflumilast occupies a large hydrophobic pocket formed by 2 Met, 1 Phe, and 1 Ser residues (Figure 5f) (Card et al, 2004). This pocket fits the 3-cyclopentyloxy group of piclamilast very well and better than 3-cyclopropylmethoxy group (Figure 6) (Card et al, 2004). The 4-difluoromethyoxy group of roflumilast also occupies a tight hydrophobic pocket with no apparent interactions for the 2 F groups (Figure 5g). The remaining interactions of both roflumilast and piclamilast with PDE4B and PDE4D are identical (Figure 6). In each complex, there are 2 monomers in the crystallographic asymmetric unit. Four copies of both roflumilast and piclamilast complexes in the 2 monomers of the 2 PDE4 structures (PDE4B and PDE4D) have nearly identical binding modes (Figure 6) (Card et al, 2004). The 3-cyclopentyloxy group of piclamilast in 2 monomers of the 1xm4 PDE4B structure was built differently in orthogonal geometry (Card et al, 2004). However, an examination of their electron density features of the deposited maps clearly showed that they were in the identical conformation. This issue can be addressed in the future using molecular dynamics (MD) simulations as to whether this group is indeed flipping or not in MD trajectories (Wang et al, 2022c).

Although apremilast also belongs to the same family of benzamides, its tail attachment that interacts with the catalytic metal ions differs from those of roflumilast and piclamilast. The distance (7.1 Å ) between the amide of acetamide group of apremilast and its core phenyl ring is very similar to the distance (6.5 Å ) of 1-amine of roflumilast (Figure 2b and c). This difference approximately matches the variation in the phosphate position between the AMP and cAMP complexes so that one might expect that the acetamide group may occupy the phosphate position of AMP. By this line of rationale, apremilast could be a product-analog inhibitor, whereas roflumilast would be a substrate-analog inhibitor. However, structure determination of the apremilast/PDE4 complex shows otherwise.

**Figure 7.**
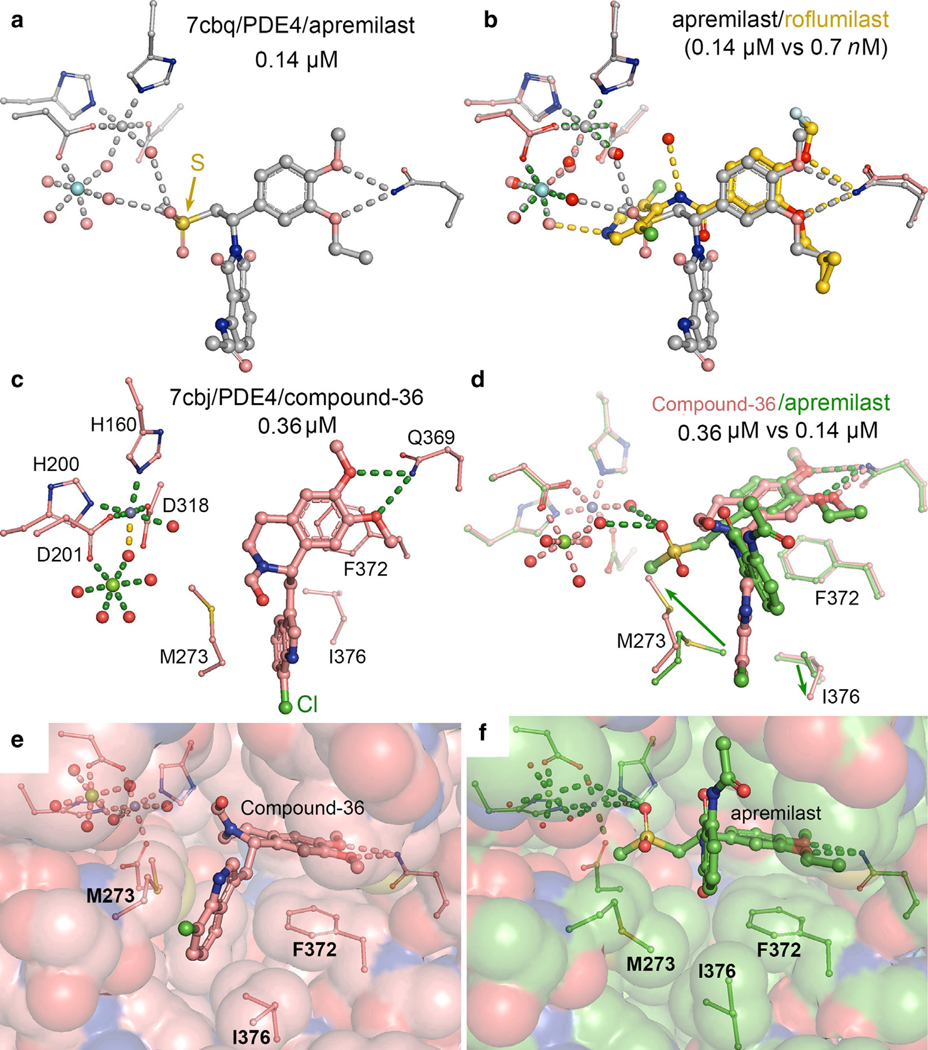
Structural comparison of apremilast, roflumilast, and compound-36 with measured IC_50_ values indicated. (**a**) Unexpected binding mode of apremilast in the active site of PDE4 where isoindole ring does not involve significant binding. Sulphur atom is shown in gold in this panel. (**b**) Superposition of apremilast (silver) with roflumilast (gold)-bound PDE4 complex reveals that isoindole ring has no counterpart in roflumilast and cAMP because roflumilast and cAMP are overlapped well as shown Figure 2. (**c**) Compound-36 complex modified from apremilast after removal of methylsulfonyl groups and making the middle appendage more rigid, repositions the sidechains of both M273 and I376 to generate a hydrophobic cavity in PDE4. (**d**) Comparison of compound-36 (salmon) with apremilast (green) within the complexes. Green arrows indicate the movements of M273 and I376 sidechains to generate a hydrophobic binding pocket in PDE4. (**e**) Surface representation of PDE4 in the compound-36/PDE4 complex. (**f**) Surface representation of PDE4 in the apremilast/PDE-4 complex. IC_50_, half-maximal inhibitory concentration; PDE4, phosphodiesterase-IV.

After structure determination of the corresponding complex, the entire isoindoyl ring of apremilast is found unexpectedly to swing out of the catalytic site and does not make any hydrophobic interactions or HBs with the enzyme (Figure 7a), with 1 exception that 1 exocyclic oxygen atom on the isoindole ring is within 3.2 Å to the F340 sidechain (unfavorable Van der Waals contacts) and 3.1 Å to an ordered water molecule for a HB (inhibitor designs are presented below) (Zhang et al, 2021). Instead, the much smaller 2-(methylsulfonyl)ethyl group of apremilast rotates into the catalytic site, with a large solvent cavity remaining. One of its sulfonyl O makes 1 set of bifurcated HBs with 2 water molecules that are ligands of both divalent metal ions. This structure explains why apremilast exhibits ~200-fold weaker inhibition of PDE4s than roflumilast; namely, the added bifurcated HBs with the methylsulfonyl O group of apremilast do not provide significant contribution to apremilast’s binding affinity to PDE4.

Extensive modifications were made after removal of methylsulfonyl group of apremilast to make its 2 rings more rigid. One such derivative was called compound-36, with improved potency comparable with that of the parental compound apremilast in inhibition of PDE4 (Figure 2) (Zhang et al, 2021). The PDE4/compound-36 complex was crystallized, and its structure was determined (Figure 7c) (Zhang et al, 2021). The structure revealed that a hydrophobic pocket was created to bind the rigid ring of compound-36 (Figure 7e) (Zhang et al, 2021), which was not present in the PDE4/apremilast complex, or in the apo structure or in many other inhibitor complexes. Therefore, PDE4s exhibit some limited plasticity that can accommodate certain ring structures attached to modified core phenyl ring. The rigid 2-ring structure of compound-36 forces the enzyme to open a new cleft for binding it but also trapping it with an enhanced high affinity, which the nonrigid 2-ring structure of apremilast failed to do (Figure 7) (Zhang et al, 2021). It is possible that a flexible linker with variable length between the 2 rings of apremilast could be used to explore for improved inhibition ability. A flexible linker is often used for design of bifunctional inhibitors to target 2 different proteins or parts of the same protein. For all these modifications, experimental data can be correlated with in silico computational studies (Maschietto et al, 2023), which will enhance the power of rational drug design.

### Structure of the PDE4/crisaborole complex, a phosphate mimic

**Figure 8.**
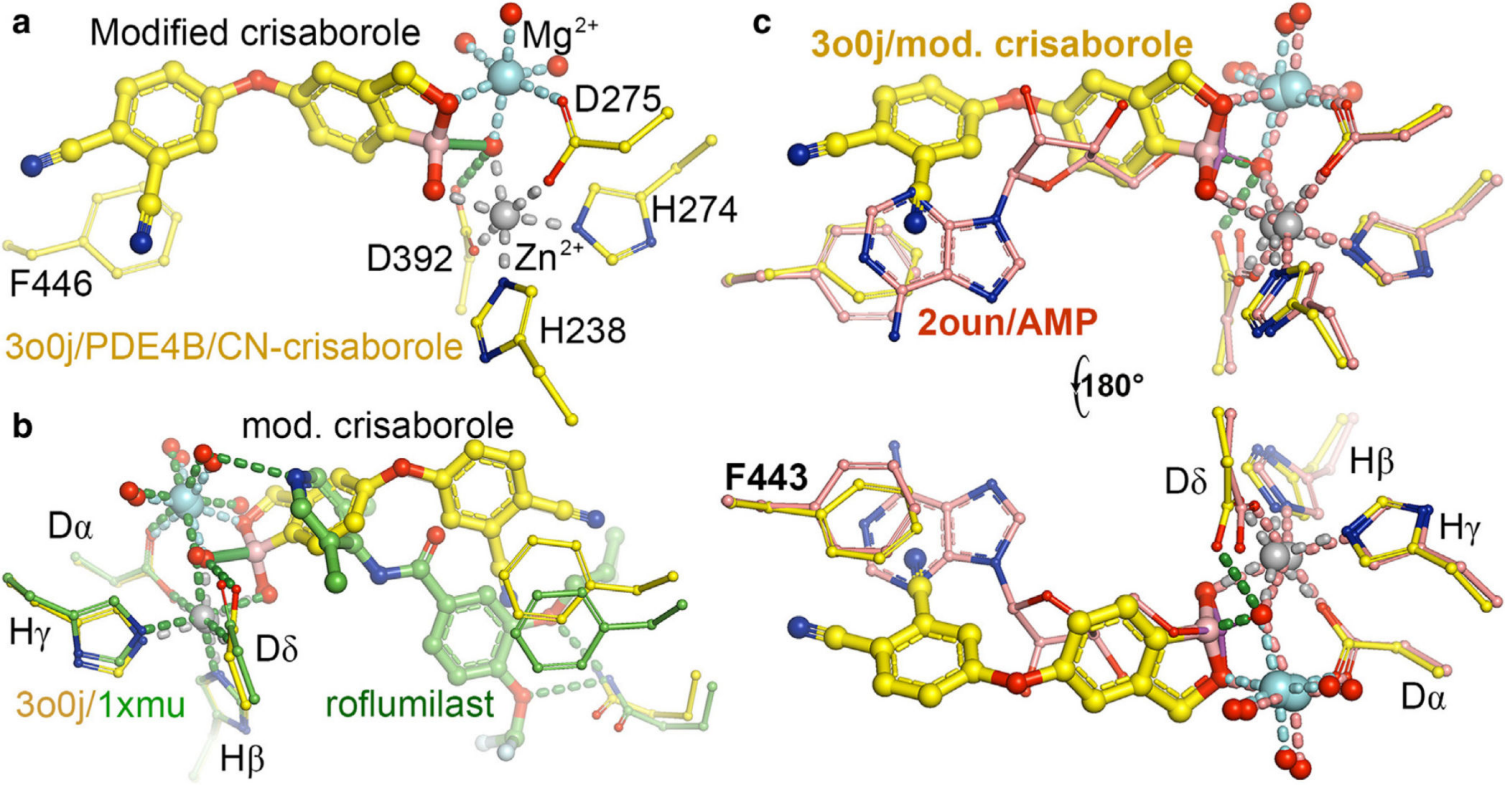
Comparison of crisaborole with roflumilast within the PDE4 complexes. (**a**) The modified crisaborole–PDE4 complex. Green bond with the boron center is the bridging μ-oxo between the 2 metal ions. (**b**) Comparison of the modified crisaborole with roflumilast within the PDE4 complexes. (**c**) Two views (front and rear) of superposition of the 3o0j (crisaborole)/2oun (AMP) structures. AMP, adenosine monophosphate; PDE4, phosphodiesterase-IV.

The secondary boron atom (ie, boron with 2 bonded atoms) can assume a trigonal planar geometry within a ring structure as a neutral species (Adamczyk-Woźniak et al, 2015; Nocentini et al, 2018; Zhang et al, 2013). The tertiary boron atom (ie, with 3 bonded atoms) can adopt a tetrahedral configuration of zwitterion with 3 O atoms, which is an ideal phosphate mimic (Figure 2d). Different from many ionic phosphate mimics, which have membrane permeability problems, benzoxaborole-based compounds do not have this problem. Unsubstituted benzoxaborole is formally named as 1,3-dihydro-1-hydroxyl-2,1-benzoxaborole, which is a distinct branch of chemistry for exploring biological functions. Crisaborole is 5-(4-cyanophenoxyl) substituted benzoxaborole and exhibits an inhibition constant of 0.75 μM for PDE4 (Figure 2). When an extra cyano group is added to the cyanophenoxyl ring of crisaborole to become 5-(3,4-dicyanophenoxyl)–substituted benzoxaborole, the inhibition ability improves by 3-fold (Figure 2h). This modified benzoxaborole was cocrystallized with PDE4 for structure determination (Figure 8) (Zhang et al, 2013). However, when boron in crisaborole was replaced with carbon, the inhibition ability for PDE4 reduced >10,000-fold, highlighting the importance of tetrahedral geometry of the boron center (Figure 2) (Zhang et al, 2013).

The modified crisaborole complex structure shows that 3 O atoms attached to boron of crisaborole become 3 O ligands to the 2 divalent metal ions, including 1 bridging μ-oxo (Figure 8a) (Zhang et al, 2013). Although boron in crisaborole is a good phosphate analog, the location of this boron within the 5-membered ring differs from the cyclic phosphate position in cAMP. If one could move the boron position by 1 atom within the 5-membered ring, it could become a better analog of the cyclic phosphate in cAMP. The consequence of the incorrectly positioned boron in crisaborole is that the remaining parts of the 2 compounds are not superimposed. The center of cyanophenoxy ring was displaced by 5.0 Å relative to the center of the core phenyl ring of roflumilast owing to different orientations of the 2 rings relative to the metal ion–binding site. Thus, crisaborole’s cyanophenoxy ring does not stack with the F446 phenyl ring (Zhang et al, 2013). When a second cyano group is added to phenoxyl ring, the newly added cyano group stacks better with the F466 ring after the phenyl ring is displaced toward it by 1.8 Å. This explains why 3,4-dicyanophenoxy–substituted benzoxaborole improves inhibition by 3-fold relative to parental crisaborole (Zhang et al, 2013). Therefore, the full inhibition ability of the parental crisaborole is solely contributed by the boron-centered phosphate mimic (Zhang et al, 2013). This explains why replacing this boron atom with a carbon atom causes the compound to lose nearly all its inhibitory ability because its hydrophobic tail attachment does not make any significant contribution to the binding affinity.

### Looking into the future: proposals for innovative designs of PDE4-specific inhibitors

Piclamilast was discovered as a potent PDE4 inhibitor before roflumilast and also exhibits a tighter binding to PDE4 (~0.03 nM) than roflumilast (0.7 nM) by 20-fold (Table 1). However, roflumilast exhibits much better selectivity against PDE4 among all human PDEs tested than piclamilast (Table 1), for which roflumilast may exhibit fewer side effects than piclamilast. Roflumilast was among the first approved for treatment of chronic obstructive pulmonary disease and has now been approved by the FDA for topical treatment of psoriasis, seborrheic dermatitis, and atopic dermatitis, but there is no information on piclamilast for any clinical trials. Apremilast is a much poorer inhibitor (140 nM) of PDE4 than roflumilast (0.7 nM) by 200-fold, and so is crisaborole (750 nM) by 1000-fold, which makes them less effective than roflumilast against PDE4. Because crisaborole recognizes mainly 2 metal ions bound in the active site as a cyclic phosphate mimic, it could target any other PDEs or even unrelated 2-metal-ion enzymes, severely limiting its selectivity against PDE4. Modifications of this compound with added specificity for PDE4 are a high priority for it to become a more effective drug. The next generation of PDE4-specific inhibitors must possess both the highest activity and the highest specificity of inhibition to this enzyme so that minimal dose of active ingredient can be used to minimize side effects. We highlight below a few proposals on how to improve the specificity of roflumilast, apremilast, and crisaborole derivatives against PDE4 using new information on how PDE4 differs from other PDEs on the basis of the structural and sequence conservation. They will be starting points for future studies of new drug discoveries.

**Figure 9.**
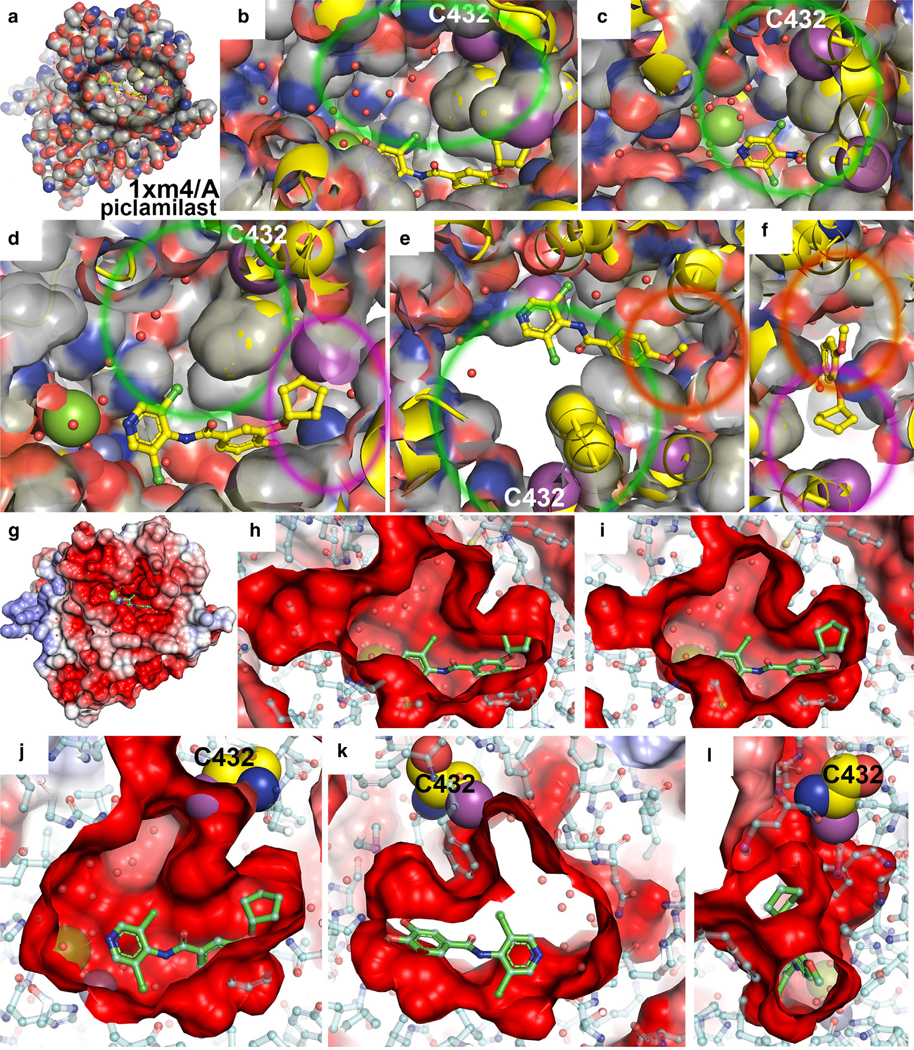
Surface representation of the inhibitor-binding pocket of the 1xm4/PDE4B piclamilast. (**a–f**) Surface colored by atom type. (**g–l**) Surface colored by electrostatic potential values. (**a, g**) Overview of monomer. (**b–f, h–l**) Close-up views of different individual subpockets. Black circle is the binding pocket in zoom-out view. Orange circles are the methoxy-binding pocket. Magenta circles are the cyclopentyloxy-binding pocket. Green circles are a large unoccupied cleft with C432 at one end of the cleft. C432 at the end of a large unoccupied cleft is shown in sphere models. Supplementary Figure S1 shows additional 3 solid electrostatic potential surface views. PDE4B, phosphodiesterase-IV B.

Starting with a surface representation of the PDE4–piclamilast complex (1×m4) (Card et al, 2004), we observe 3 distinct pockets where we propose to modify existing inhibitors (Figures 2 and 9 and Supplementary Figure S1): (i) a large unoccupied pocket in both roflumilast and piclamilast and a partially occupied pocket in apremilast, (ii) position-4 of the benzamide where it is a methoxy group in piclamilast and apremilast but a difluoromethoxy group in roflumilast, and (iii) position-3 of the benzamide where it is an ethanoxy group in apremilast, cyclopropyloxy in roflumilast, and cyclopentyloxy in piclamilast.

**Figure 10.**
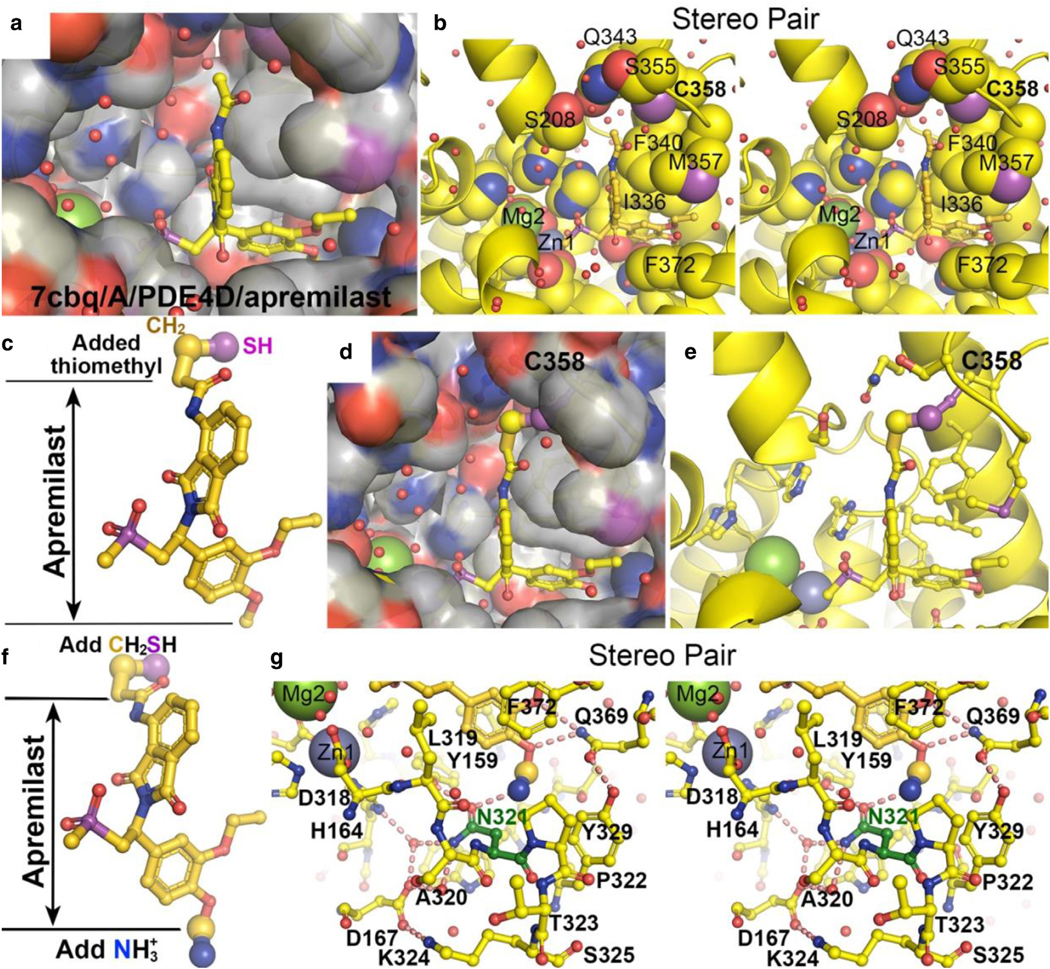
Proposed modifications of apremilast to become more specific, higher-potent PDE4 inhibitors. (**a**) Surface representation of the apremilast-binding pocket. (**b**) Stereo pair of the binding pocket with key residues shown in large spheres and labeled, including the C358 sidechain. (**c**) A proposed modification to apremilast by insertion of thiomethyl group at the end of the acetamide C atom, which places the thiol group within disulfide bond distance of C358 for a covalent inhibitor. (**d, e**) Proposed disulfide bond (**d**) with and (**e**) without surface representation. (**f**) A second proposed insertion of a primary amine at C atom of 4-methoxy of benzamide. (**g**) Stereodiagram of modified compound forms a HB with the N321 sidechain (shown in green) in the binding pocket of this substitution. This view also shows how Q369 is oriented by Y329. HB, hydrogen bond; PDE4, phosphodiesterase-IV.

In the apremilast/PDE4D (7cbq) complex (Zhang et al, 2021), the isoindoyl ring of apremilast occupies partially a large pocket but does not bind it favorably. It makes only a single HB but also makes some unfavorable hydrophilic–hydrophobic contacts. Therefore, it remains unclear whether it contributes positive or negative to its binding affinity. At the end of this pocket, there is an exposed Cys sidechain in PDE4 structures, which often formed a disulfide bond with β-mercaptoethanol when it was present as an additive in crystallization solution but not in the apremilast/PDE4D (7cbq) complex. In this complex, the acetamide group points to C358, which is C432 in the PDE4B/1xm4 structure (Figure 9) (Card et al, 2004). This Cys residue is present in all PDE4 isoforms and in PDE1B and PDE7B but not in other PDEs (Figure 3). When a thiomethyl group is computationally grafted onto C atom of the acetamide of apremilast, the added thiol group is within the ideal distance for formation of a disulfide bond with C358 (Figure 10c–e). Such a covalent inhibitor would be highly specific to these 4 particular PDEs and irreversibly inhibit them by forming dead-end covalently inhibited complexes. It is noted that 1 of the most potent anti–severe acute respiratory syndrome coronavirus 2 main protease inhibitors, Paxlovid, is also a covalent inhibitor (La Monica et al, 2022; Marzi et al, 2022). Other small molecules containing cysteine-reactive “warhead” include Ebselen (Capper et al, 2018).

In the position-4 pocket of benzamide, fluorine atoms of the difluoromethoxy group of roflumilast do not interact favorably with any PDE residue. In this pocket, N321 adopts the most preferred rotamer conformation, as in almost all PDE complexes with other inhibitors and in the apo PDE4 structures (Figure 10f and g). In this conformation, N321 forms HBs with the invariant Y159 residue and 2 ordered water molecules that are shared with D167 (Figure 10). In this orientation, the difluoride substitutions of roflumilast rotate away from the N321 sidechain carbonyl group relative to one in the PDE4/AMP or PDE4/cAMP complexes, in the latter of which N321 adopts a rotamer that is closer to the fourth favorable rotamer and recognizes both N7 and N6 of the adenine ring of cAMP and AMP. In this sense, absence of these 2 difluoride substitutions on apremilast should be a positive contribution to its binding affinity relative to roflumilast. We propose to insert a primary amine to the 4-methoxy group into apremilast (Figure 10f). According to our modeling, such insertion would result in a favorable HB between the added amine and the N321 sidechain carbonyl group (Figure 10g). Given that this Asn residue is mainly present in PDE4 isoforms and other 2 cAMP-specific PDEs (Table 1), this insertion would also improve the selectivity for these enzymes. This modification can be done to both roflumilast and piclamilast and all other related benzamide-based PDE4 inhibitors.

The size of the position-3 pocket is quite large, which is actually larger than the cyclopentyloxy group of piclamilast (Figure 9). As such, the binding affinity for the group at this pocket should increase gradually and the total affinity when it increases size from an ethanoxy, cyclopropyloxy, to cyclopentyloxy group. Therefore, we can increase the binding affinity and enzyme selectivity of proposed derivatives at this site because the hydrophobic surface (ΔS_Area_) buried between the enzyme and an inhibitor increases with increasing size of the substituent and provides free energy for binding, that is, ΔG = -RTln[*K*_d_] α ΔS_Area_.

**Figure 11.**
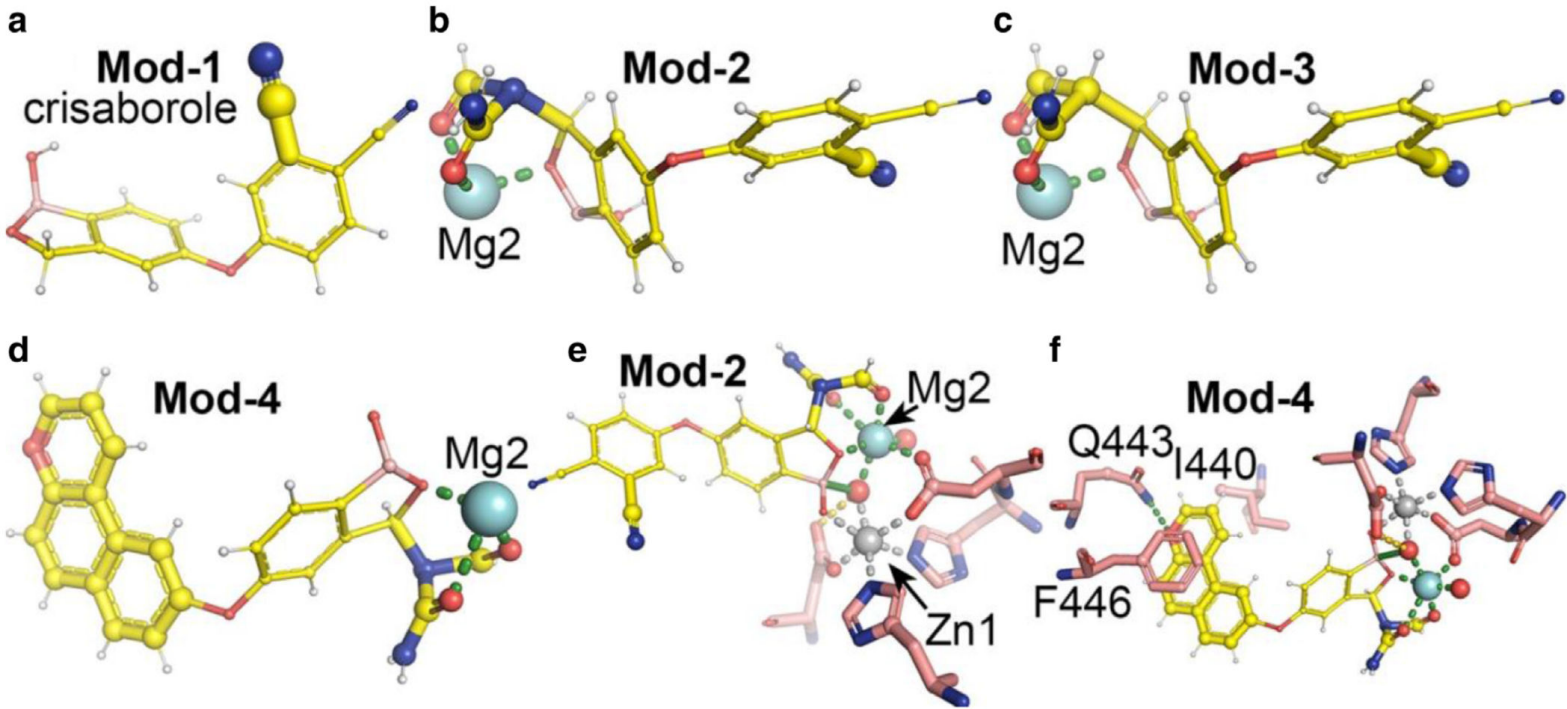
Modified crisaborole and proposed other modifications. (**a**) An extra cyano substitution to the phenolic ring to increase its binding affinity in Mod-1, whose complex structure was known. (**b**) Second modification (Mod-2): an N(CHO)(C-CONH_2_) group attached with a chiral center to the borole ring for binding of Mg^2+^ ion corresponding to the second metal ionebinding site (Mg2). (**c**) Mod-3: a CH(CHO)(C-CONH_2_) attachment. (**d**) Mod-4: an attachment of a large hydrophobic ring with one functional group within the ring for formation of HB to Q443 after additional Mod-2. (**e**) Modeled complex of the Mod-2 of crisaborole with PDE4B. (**f**) Modeled complex of the Mod-4 of crisaborole with PDB4B. Location of the functional -O- group within the attached ring may need to be repositioned. HB, hydrogen bond; Mod-1, modification #1; Mod-2, modification #2; Mod-3, modification #3; Mod-4, modification #4; PDE4B, phosphodiesterase-IV B.

Crisaborole is a mimic of the cyclic phosphate of cAMP. Its binding mode is unrelated to that of apremilast and other benzamides. It is neither involved in the Q443 sidechain for recognition of N1 of cAMP and AMP nor stacking interactions with 1 Phe and an Ile sidechain. We propose 3 modifications to crisaborole in addition to one that has already been made for which the complex structure with PDB4B has been determined (Figure 11) (Zhang et al, 2013). In this complex structure, the added cyano group stacks with the F446 side-chain after F446 is displaced toward it for maximal interactions. Proposed modification #1 is to insert an N(CHO)(C-CONH_2_) group to provide 2 additional O ligands for chelating Mg^2+^ ion where 2 O ligands occupy approximately the same locations of 2 of the 5 internally bound ordered water molecules in the PDE structures (Figure 11b). Proposed modification #2 is to replace the ternary amine with a carbon atom, that is, a CH(CHO)(C-CONH_2_) group (Figure 11c). These 2 modifications have different hydrophobicity. With 2 added HBs from the 2 modifications, binding affinity of modified inhibitors should increase greatly, particularly because 2 replaced water molecules are part of extension arms of invariant sidechains of the PDEs (Figure 11b and c). Proposed modification #3 is to extend aromatic surface to stack with the F446 and I440 sidechains, which was missing on the original crisaborole (Figure 11d). In addition, a properly positioned HB acceptor, whose position is only approximately estimated, with Q443 could further increase binding affinity as in roflumilast and apremilast (Figure 11).

### Machine learning and artificial intelligence in inhibitor designs

Chemical, biochemical, and structural databases for PDE4 inhibitors have rapidly accumulated for the past 3 decades to become among the largest knowledge databases. We still envision numerous opportunities for designing the next generation of inhibitors, likely more powerful and more specific. Earlier, we provided a few examples of inhibitor designs, targeting the invariant Gln residue and PDE4-specific Asn residue for recognition of both AMP and cAMP, 2 divalent metal ions and 5 internal water molecules W1–W5, and the PDE4-specific Cys residue. Currently, we propose 1 design at a time. We can also use combinatory methods to properly integrate them in a cooperative manner. When combining with different starting points (roflumilast or apremilast), the number of proposed inhibitor derivatives could increase exponentially. We expect to be able to rank the proposed derivatives on the basis of either binding affinity or enzyme specificity computationally before they are synthesized.

For increasing contact surface between the enzyme and inhibitor, its binding affinity will increase, that is, ΔG = -RTln[*K*_d_] α ΔS_Area_, which can be partly addressed using empirical linear least-squares fitting methods. However, both the surface and electrostatic complementarities between them are as important as the total buried surface area so that the relationship is not simply linear. This complex relationship is better addressed using machine learning and artificial intelligence. A simple version of this application was demonstrated for PDE4 inhibitors in 2013 (Niu et al, 2013). This application should include MD simulations and free energy estimation in the future.

## CONCLUDING REMARKS

The most recently FDA-approved cAMP-specific PDE4 inhibitor roflumilast has a half-maximal inhibitory concentration value of 0.7 nM, which is the strongest among dermatology-approved PDE4 inhibitors and explains why it is highly effective in its label-indicated skin diseases as well as off label in other “roflumilast-responsive dermatoses”; however, we believe that there is room for further improvement to create a next generation of PDE4-specific inhibitors. We propose that the next generation of powerful PDE4-specific inhibitors will include (i) covalent PDE4 inhibitors and (ii) crisaborole derivatives with improved PDE4 specificity for targeting only PDE4 2-metal ions. We foresee that integration of machine learning, artificial intelligence, and other computational biophysics will play a significant role in future PDE4 drug discovery given recent data demonstrating that MD simulations produce electrostatic potential maps that are highly comparable with experimental cryogenic electron microscopy maps (Wang et al, 2022a, 2022b, 2022c). In the near future, we can produce the reliable equilibrated structure of any enzyme–inhibitor complex using MD simulations and accurately predict its binding properties even before the given inhibitor is ever synthesized. This approach will revolutionize rational drug discovery and bring new state-of-art therapies into the clinic.

## MATERIALS AND METHODS

This study independently analyzed recent crystallographic and biochemical data of relevant PDE4 complexes and provides a structural basis as to why a single replacement of a boron atom in crisaborole with carbon, for example, reduces its inhibitory constant by over 5 orders of magnitude. To ensure the validity of crystallographic structures discussed in this study, all relevant atomic models alongside with the corresponding electron density maps were retrieved from the Protein Data Bank whenever available and carefully examined using the graphics program Coot (Emsley and Cowtan, 2004). Simplified modeling was carried out by taking parts from known structures, for example, a cyano group from crisaborole for grafting onto other parts of known compounds, a thiol group from Cys sidechain, an amine group from Lys sidechain, and so on, followed by geometric regulation using the program libcheck of CCP4 package. This procedure provided a reasonable view on the approximate size of added or modified groups. All graphics figures in this study are original and made using program PyMol (Schrodinger and DeLano, 2020).

## Supplementary Material

mmcjpg

mmc1

1

## Figures and Tables

**Table 1.** Reported IC_50_ Values for Inhibitors Derived from Zhang et al (2004) and *K*_M_ Values for cAMP and cGMP Derived from Bender and Beavo (2006) in μM

PDE	Roflumilast/IC_50_	Piclamilast/IC_50_	cAMP/K_M_	cGMP/K_M_

PDE1B	>200	68	10–24	1.2–5.9
PDE2A	>200	54	30	20
PDE3B	>200	11	0.38	0.28
PDE4B	0.00084	0.000041	1.5–4.7	Not bound
PDE4D	0.00068	0.000021	1.2–5.9	Not bound
PDE5A	17	3.5	290	2.9–6.2
PDE6A	Not reported	Not reported	700	15
PDE7B	>200	8.8	0.03–0.07	Not bound
PDE8A	>200	>200	0.06	Not bound
PDE9A	>200	>200	230	0.7–0.17
PDE10A	>200	21	0.22–1.1	13–14
PDE11A	25	1.6	2.0–3.2	0.95–2.1

Abbreviations: IC_50_, half-maximal inhibitory concentration; K_M_, Michaelis–Menten constant; PDE, phosphodiesterase.

## Data Availability

Structural coordinates for all experimentally determined protein and protein–ligand structures analyzed and discussed in this manuscript are provided and accessible at https://www.rcsb.org/, hosted at the Protein Data Bank (RCSB PDB).

## Abbreviations:

AC: adenylyl cyclase
AMP: adenosine monophosphate
EP: enzyme–product
FDA: Food and Drug Administration
HB: hydrogen bond
MD: molecular dynamics
PDE: phosphodiesterase
PKA: protein kinase A

## References

[R1] Adamczyk-Woźniak A, Borys KM, Sporzyński A. Recent developments in the chemistry and biological applications of benzoxaboroles. Chem Rev 2015;115:5224–47. 26017806 10.1021/cr500642d

[R2] Aravind L, Koonin EV. The HD domain defines a new superfamily of metal-dependent phosphohydrolases. Trends Biochem Sci 1998;23:469–72. 9868367 10.1016/s0968-0004(98)01293-6

[R3] Ashton MJ, Cook DC, Fenton G, Karlsson JA, Palfreyman MN, Raeburn D, Selective type IV phosphodiesterase inhibitors as antiasthmatic agents. The syntheses and biological activities of 3-(cyclopentyloxy)-4-methoxybenzamides and analogues. J Med Chem 1994;37:1696–703. 8201604 10.1021/jm00037a021

[R4] Aslam M, Ladilov Y. Emerging role of cAMP/AMPK signaling. Cells 2022;11:308. 35053423 10.3390/cells11020308 PMC8774420

[R5] Basu G, Sivanesan D, Kawabata T, Go N. Electrostatic potential of nucleotide-free protein is sufficient for discrimination between adenine and guanine-specific binding sites. J Mol Biol 2004;342:1053–66. 15342256 10.1016/j.jmb.2004.07.047

[R6] Beeley RRA, Millican TA. Trisubstituted phenyl derivatives as selective phosphodiesterase IV inhibitors 1993. https://patents.google.com/patent/DK0607373T3/en. (accessed January 16, 2023).

[R7] Beese LS, Steitz TA. Structural basis for the 3’−5’ exonuclease activity of Escherichia coli DNA polymerase I: a two metal ion mechanism. EMBO J 1991;10:25–33. 1989886 10.1002/j.1460-2075.1991.tb07917.x PMC452607

[R8] Bender AT, Beavo JA. Cyclic nucleotide phosphodiesterases: molecular regulation to clinical use. Pharmacol Rev 2006;58:488–520. 16968949 10.1124/pr.58.3.5

[R9] Boswell-Smith V, Spina D. PDE4 inhibitors as potential therapeutic agents in the treatment of COPD-focus on Roflumilast. Int J Chron Obstruct Pulmon Dis 2007;2:121–9. 18044684 PMC2695611

[R10] Calamera G, Moltzau LR, Levy FO, Andressen KW. Phosphodiesterases and compartmentation of cAMP and cGMP signaling in regulation of cardiac contractility in normal and failing hearts. Int J Mol Sci 2022;23:2145. 35216259 10.3390/ijms23042145 PMC8880502

[R11] Capper MJ, Wright GSA, Barbieri L, Luchinat E, Mercatelli E, McAlary L, The cysteine-reactive small molecule ebselen facilitates effective SOD1 maturation. Nat Commun 2018;9:1693. 29703933 10.1038/s41467-018-04114-x PMC5923229

[R12] Card GL, England BP, Suzuki Y, Fong D, Powell B, Lee B, Structural basis for the activity of drugs that inhibit phosphodiesterases. Structure 2004;12:2233–47. 15576036 10.1016/j.str.2004.10.004

[R13] Cardarelli S, Biglietto M, Orsini T, Fustaino V, Monaco L, de Oliveira do Rêgo AG, Modulation of cAMP/cGMP signaling as prevention of congenital heart defects in Pde2A deficient embryos: a matter of oxidative stress. Cell Death Dis 2024;15:169. 38395995 10.1038/s41419-024-06549-1 PMC10891154

[R14] Carlezon WA Jr, Duman RS, Nestler EJ. The many faces of CREB. Trends Neurosci 2005;28:436–45. 15982754 10.1016/j.tins.2005.06.005

[R15] Cedervall P, Aulabaugh A, Geoghegan KF, McLellan TJ, Pandit J. Engineered stabilization and structural analysis of the autoinhibited conformation of PDE4. Proc Natl Acad Sci USA 2015;112:E1414–22. 25775568 10.1073/pnas.1419906112 PMC4378417

[R16] Chiricozzi A, Caposiena D, Garofalo V, Cannizzaro MV, Chimenti S, Saraceno R. A new therapeutic for the treatment of moderate-to-severe plaque psoriasis: apremilast. Expert Rev Clin Immunol 2016;12:237–49. 26692125 10.1586/1744666X.2016.1134319

[R17] Conti M, Beavo J. Biochemistry and physiology of cyclic nucleotide phosphodiesterases: essential components in cyclic nucleotide signaling. Annu Rev Biochem 2007;76:481–511. 17376027 10.1146/annurev.biochem.76.060305.150444

[R18] Dyda F, Hickman AB, Jenkins TM, Engelman A, Craigie R, Davies DR. Crystal structure of the catalytic domain of HIV-1 integrase: similarity to other polynucleotidyl transferases. Science 1994;266:1981–6. 7801124 10.1126/science.7801124

[R19] Emsley P, Cowtan K. Coot: model-building tools for molecular graphics. Acta Crystallogr D Biol Crystallogr 2004;60:2126–32. 15572765 10.1107/S0907444904019158

[R20] Epstein PM. Different phosphodiesterases (PDEs) regulate distinct phosphoproteomes during cAMP signaling. Proc Natl Acad Sci USA 2017;114:7741–3. 28710333 10.1073/pnas.1709073114 PMC5544341

[R21] Fabbri LM, Beghé B, Yasothan U, Kirkpatrick P. Roflumilast [published correction appears in Nat Rev Drug Discov 2024;23:563]. Nat Rev Drug Discov 2010;9:761–2. 20885403 10.1038/nrd3276

[R22] Flockerzi D, Gutterer B, Hatzelmann A, Schudt C, Beume R, Kilian U, Fluoroalkoxy-substituted benzamides and their use as cyclic nucleotide phosphodiesterase inhibitors. 1995. World-wide patent WO95/01338. In: WO95. W-wp.

[R23] Freund YR, Akama T, Alley MR, Antunes J, Dong C, Jarnagin K, Boron-based phosphodiesterase inhibitors show novel binding of boron to PDE4 bimetal center. FEBS Lett 2012;586:3410–4. 22841723 10.1016/j.febslet.2012.07.058

[R24] Gaudin C, Homcy CJ, Ishikawa Y. Mammalian adenylyl cyclase family members are randomly located on different chromosomes. Hum Genet 1994;94:527–9. 7959689 10.1007/BF00211020

[R25] Goldgur Y, Craigie R, Cohen GH, Fujiwara T, Yoshinaga T, Fujishita T, Structure of the HIV-1 integrase catalytic domain complexed with an inhibitor: a platform for antiviral drug design. Proc Natl Acad Sci USA 1999;96:13040–3. 10557269 10.1073/pnas.96.23.13040 PMC23896

[R26] Hanoune J, Defer N. Regulation and role of adenylyl cyclase isoforms. Annu Rev Pharmacol Toxicol 2001;41:145–74. 11264454 10.1146/annurev.pharmtox.41.1.145

[R27] Hardie DG. AMP-activated protein kinase: a cellular energy sensor with a key role in metabolic disorders and in cancer. Biochem Soc Trans 2011;39:1–13. 10.1042/BST0390001 21265739

[R28] Hauser AS, Attwood MM, Rask-Andersen M, Schiöth HB, Gloriam DE. Trends in GPCR drug discovery: new agents, targets and indications. Nat Rev Drug Discov 2017;16:829–42. 29075003 10.1038/nrd.2017.178 PMC6882681

[R29] Hoffmann R, Baillie GS, MacKenzie SJ, Yarwood SJ, Houslay MD. The MAP kinase ERK2 inhibits the cyclic AMP-specific phosphodiesterase HSPDE4D3 by phosphorylating it at Ser579. EMBO J 1999;18:893–903. 10022832 10.1093/emboj/18.4.893 PMC1171182

[R30] Huai Q, Colicelli J, Ke H. The crystal structure of AMP-bound PDE4 suggests a mechanism for phosphodiesterase catalysis. Biochemistry 2003;42:13220–6. 14609333 10.1021/bi034653e

[R31] Insel PA, Sriram K, Gorr MW, Wiley SZ, Michkov A, Salmerón C, GPCRomics: an approach to discover GPCR drug targets. Trends Pharmacol Sci 2019;40:378–87. 31078319 10.1016/j.tips.2019.04.001 PMC6604616

[R32] Issa NT, Byers SW, Dakshanamurthy S. ES-screen: a novel electrostatics-driven method for drug discovery virtual screening. Int J Mol Sci 2022;23:14830. 36499162 10.3390/ijms232314830 PMC9736079

[R33] Jansen C, Kooistra AJ, Kanev GK, Leurs R, de Esch IJ, de Graaf C. PDEStrIAn: A phosphodiesterase structure and ligand interaction annotated database as a tool for structure-based drug design. J Med Chem 2016;59:7029–65. 26908025 10.1021/acs.jmedchem.5b01813

[R34] Joshi T, Yan D, Hamed O, Tannheimer SL, Phillips GB, Wright CD, GS-5759, a bifunctional β2-Adrenoceptor agonist and phosphodiesterase 4 inhibitor for chronic obstructive pulmonary disease with a unique mode of action: effects on gene expression in human airway epithelial cells. J Pharmacol Exp Ther 2017;360:324–40. 27927912 10.1124/jpet.116.237743

[R35] Keravis T, Lugnier C. Cyclic nucleotide phosphodiesterase (PDE) isozymes as targets of the intracellular signalling network: benefits of PDE inhibitors in various diseases and perspectives for future therapeutic developments. Br J Pharmacol 2012;165:1288–305. 22014080 10.1111/j.1476-5381.2011.01729.x PMC3372715

[R36] Kraut J. How do enzymes work? Science 1988;242:533–40. 3051385 10.1126/science.3051385

[R37] La Monica G, Bono A, Lauria A, Martorana A. Targeting SARS-CoV-2 main protease for treatment of COVID-19: covalent inhibitors structure-activity relationship insights and evolution perspectives. J Med Chem 2022;65:12500–34. 36169610 10.1021/acs.jmedchem.2c01005 PMC9528073

[R38] Laskowski RA, Macarthur MW, Moss DS, Thornton JM. PROCHECK: a program to check the stereochemical quality of protein structures. J Appl Crystallogr 1993;26:283–91.

[R39] Li H, Zuo J, Tang W. Phosphodiesterase-4 inhibitors for the treatment of inflammatory diseases. Front Pharmacol 2018;9:1048. 30386231 10.3389/fphar.2018.01048 PMC6199465

[R40] Liu S, Mansour MN, Dillman KS, Perez JR, Danley DE, Aeed PA, Structural basis for the catalytic mechanism of human phosphodiesterase 9. Proc Natl Acad Sci USA 2008;105:13309–14. 18757755 10.1073/pnas.0708850105 PMC2533186

[R41] MacKenzie SJ, Baillie GS, McPhee I, MacKenzie C, Seamons R, McSorley T, Long PDE4 cAMP specific phosphodiesterases are activated by protein kinase A-mediated phosphorylation of a single serine residue in upstream conserved Region 1 (UCR1). Br J Pharmacol 2002;136:421–33. 12023945 10.1038/sj.bjp.0704743 PMC1573369

[R42] Manganiello V. Short-term regulation of PDE4 activity. Br J Pharmacol 2002;136:339–40. 12023934 10.1038/sj.bjp.0704741 PMC1573368

[R43] Martinez SE, Beavo JA, Hol WG. GAF domains: two-billion-year-old molecular switches that bind cyclic nucleotides. Mol Interv 2002a;2:317–23. 14993386 10.1124/mi.2.5.317

[R44] Martinez SE, Wu AY, Glavas NA, Tang XB, Turley S, Hol WG, The two GAF domains in phosphodiesterase 2A have distinct roles in dimerization and in cGMP binding. Proc Natl Acad Sci USA 2002b;99:13260–5. 12271124 10.1073/pnas.192374899 PMC130621

[R45] Marzi M, Vakil MK, Bahmanyar M, Zarenezhad E. Paxlovid: mechanism of action, synthesis, and in silico study. BioMed Res Int 2022;2022:7341493. 35845944 10.1155/2022/7341493 PMC9283023

[R46] Maschietto F, Qiu T, Wang J, Shi Y, Allen B, Lisi GP, Valproate-coenzyme A conjugate blocks opening of receptor binding domains in the spike trimer of SARS-CoV-2 through an allosteric mechanism. Comput Struct Biotechnol J 2023;21:1066–76. 36688026 10.1016/j.csbj.2023.01.014 PMC9841741

[R47] Myeku N, Clelland CL, Emrani S, Kukushkin NV, Yu WH, Goldberg AL, Tau-driven 26S proteasome impairment and cognitive dysfunction can be prevented early in disease by activating cAMP-PKA signaling. Nat Med 2016;22:46–53. 26692334 10.1038/nm.4011 PMC4787271

[R48] Niu M, Dong F, Tang S, Fida G, Qin J, Qiu J, Pharmacophore modeling and virtual screening for the discovery of new type 4 cAMP phosphodiesterase (PDE4) inhibitors. PLoS One 2013;8:e82360. 24340020 10.1371/journal.pone.0082360 PMC3858292

[R49] Nocentini A, Supuran CT, Winum JY. Benzoxaborole compounds for therapeutic uses: a patent review (2010– 2018). Expert Opin Ther Pat 2018;28:493–504. 29727210 10.1080/13543776.2018.1473379

[R50] Omori K, Kotera J. Overview of PDEs and their regulation. Circ Res 2007;100:309–27. 17307970 10.1161/01.RES.0000256354.95791.f1

[R51] Ostrom KF, LaVigne JE, Brust TF, Seifert R, Dessauer CW, Watts VJ, Physiological roles of mammalian transmembrane adenylyl cyclase isoforms. Physiol Rev 2022;102:815–57. 34698552 10.1152/physrev.00013.2021 PMC8759965

[R52] Paes D, Schepers M, Rombaut B, van den Hove D, Vanmierlo T, Prickaerts J. The molecular biology of phosphodiesterase 4 enzymes as pharmacological targets: an interplay of isoforms, conformational states, and inhibitors. Pharmacol Rev 2021;73:1016–49. 34233947 10.1124/pharmrev.120.000273

[R53] Pagès L, Gavaldà A, Lehner MD. PDE4 inhibitors: a review of current developments (2005 – 2009). Expert Opin Ther Pat 2009;19:1501–19. 19832118 10.1517/13543770903313753

[R54] Russell FM, Hardie DG. AMP-activated protein kinase: do we need activators or inhibitors to treat or prevent cancer? Int J Mol Sci 2020;22:186. 33375416 10.3390/ijms22010186 PMC7795930

[R55] Sakkas LI, Mavropoulos A, Bogdanos DP. Phosphodiesterase 4 inhibitors in immune-mediated diseases: mode of action, clinical applications, current and future perspectives. Curr Med Chem 2017;24:3054–67. 28554321 10.2174/0929867324666170530093902

[R56] Schafer PH, Parton A, Capone L, Cedzik D, Brady H, Evans JF, Apremilast is a selective PDE4 inhibitor with regulatory effects on innate immunity. Cell Signal 2014;26:2016–29. 24882690 10.1016/j.cellsig.2014.05.014

[R57] Schrodinger L, DeLano W. PyMol. http://www.pymol.org/; 2020. (accessed September 2, 2022).

[R58] Seifert R, Lushington GH, Mou TC, Gille A, Sprang SR. Inhibitors of membranous adenylyl cyclases. Trends Pharmacol Sci 2012;33:64–78. 22100304 10.1016/j.tips.2011.10.006 PMC3273670

[R59] Shaywitz AJ, Greenberg ME. CREB: a stimulus-induced transcription factor activated by a diverse array of extracellular signals. Annu Rev Biochem 1999;68:821–61. 10872467 10.1146/annurev.biochem.68.1.821

[R60] Smith SJ, Zhao XZ, Passos DO, Lyumkis D, Burke TR Jr, Hughes SH. Integrase strand transfer inhibitors are effective anti-HIV drugs. Viruses 2021;13. 33572956 10.3390/v13020205 PMC7912079

[R61] Sriram K, Insel PA. G protein-coupled receptors as targets for approved drugs: how many targets and how many drugs? Mol Pharmacol 2018;93:251–8. 29298813 10.1124/mol.117.111062 PMC5820538

[R62] Steitz TA. DNA polymerases: structural diversity and common mechanisms. J Biol Chem 1999;274:17395–8. 10364165 10.1074/jbc.274.25.17395

[R63] Steitz TA, Steitz JA. A general two-metal-ion mechanism for catalytic RNA. Proc Natl Acad Sci USA 1993;90:6498–502. 8341661 10.1073/pnas.90.14.6498 PMC46959

[R64] Sunahara RK, Dessauer CW, Gilman AG. Complexity and diversity of mammalian adenylyl cyclases. Annu Rev Pharmacol Toxicol 1996;36:461–80. 8725398 10.1146/annurev.pa.36.040196.002333

[R65] Thompson JD, Higgins DG, Gibson TJ. Clustal W: improving the sensitivity of progressive multiple sequence alignment through sequence weighting, position-specific gap penalties and weight matrix choice. Nucleic Acids Res 1994;22:4673–80. 7984417 10.1093/nar/22.22.4673 PMC308517

[R66] Wang H, Liu Y, Hou J, Zheng M, Robinson H, Ke H. Structural insight into substrate specificity of phosphodiesterase 10. Proc Natl Acad Sci USA 2007a;104:5782–7. 17389385 10.1073/pnas.0700279104 PMC1851569

[R67] Wang H, Robinson H, Ke H. The molecular basis for different recognition of substrates by phosphodiesterase families 4 and 10. J Mol Biol 2007b;371:302–7. 17582435 10.1016/j.jmb.2007.05.060 PMC2001251

[R68] Wang J, Bunick CG. 1130 Clinically relevant differences in the chemical and structural mechanism of action of dermatological phosphodiesterase-4 inhibitors. J Invest Dermatol 2023;143:S194 [abstr.].

[R69] Wang J, Shi Y, Reiss K, Allen B, Maschietto F, Lolis E, Insights into binding of single-stranded viral RNA template to the replication-transcription complex of SARS-CoV-2 for the priming reaction from Molecular Dynamics simulations. Biochemistry 2022a;61:424–32. 35199520 10.1021/acs.biochem.1c00755

[R70] Wang J, Shi Y, Reiss K, Maschietto F, Lolis E, Konigsberg WH, Structural insights into binding of remdesivir triphosphate within the replication-transcription complex of SARS-CoV-2. Biochemistry 2022b;61:1966–73. 36044776 10.1021/acs.biochem.2c00341 PMC9469760

[R71] Wang J, Skeens E, Arantes PR, Maschietto F, Allen B, Kyro GW, Structural basis for reduced dynamics of three engineered HNH endonuclease Lys-to-Ala mutants for the clustered regularly interspaced short palindromic repeat (CRISPR)-associated 9 (CRISPR/Cas9) enzyme. Biochemistry 2022c;61:785–94. 35420793 10.1021/acs.biochem.2c00127 PMC9069930

[R72] Xie M, Blackman B, Scheitrum C, Mika D, Blanchard E, Lei T, The upstream conserved regions (UCRs) mediate homo- and hetero-oligomerization of type 4 cyclic nucleotide phosphodiesterases (PDE4s). Biochem J 2014;459:539–50. 24555506 10.1042/BJ20131681 PMC4315173

[R73] Yang D, Zhou Q, Labroska V, Qin S, Darbalaei S, Wu Y, G protein-coupled receptors: structure- and function-based drug discovery. Signal Transduct Target Ther 2021;6:7. 33414387 10.1038/s41392-020-00435-w PMC7790836

[R74] Zaccolo M, Movsesian MA. cAMP and cGMP signaling cross-talk: role of phosphodiesterases and implications for cardiac pathophysiology. Circ Res 2007;100:1569–78. 17556670 10.1161/CIRCRESAHA.106.144501

[R75] Zaccolo M, Zerio A, Lobo MJ. Subcellular organization of the cAMP signaling pathway. Pharmacol Rev 2021;73:278–309. 33334857 10.1124/pharmrev.120.000086 PMC7770493

[R76] Zhang J, Zhu MY, Lin YN, Zhou HC. The synthesis of benzoxaboroles and their applications in medicinal chemistry. Sci China Chem 2013;56:1372–81.

[R77] Zhang KY, Card GL, Suzuki Y, Artis DR, Fong D, Gillette S, A glutamine switch mechanism for nucleotide selectivity by phosphodiesterases. Mol Cell 2004;15:279–86. 15260978 10.1016/j.molcel.2004.07.005

[R78] Zhang R, Li H, Zhang X, Li J, Su H, Lu Q, Design, synthesis, and biological evaluation of tetrahydroisoquinolines derivatives as novel, selective PDE4 inhibitors for antipsoriasis treatment. Eur J Med Chem 2021;211:113004. 33218684 10.1016/j.ejmech.2020.113004

[R79] Zhu H, Suk HY, Yu RY, Brancho D, Olabisi O, Yang TT, Evolutionarily conserved role of calcineurin in phosphodegron-dependent degradation of phosphodiesterase 4D. Mol Cell Biol 2010;30:4379–90. 20647544 10.1128/MCB.01193-09 PMC2937537

[R80] Zuo H, Cattani-Cavalieri I, Musheshe N, Nikolaev VO, Schmidt M. Phosphodiesterases as therapeutic targets for respiratory diseases. Pharmacol Ther 2019;197:225–42. 30759374 10.1016/j.pharmthera.2019.02.002

