# Supplementary figures and images for: Chemical, Biochemical, and Structural Similarities and Differences of Dermatological cAMP Phosphodiesterase-IV Inhibitors
Source: J Invest Dermatol. Author manuscript; Available in PMC 2025 Jun 1. (PMC12103293; doi:10.1016/j.jid.2024.10.597)

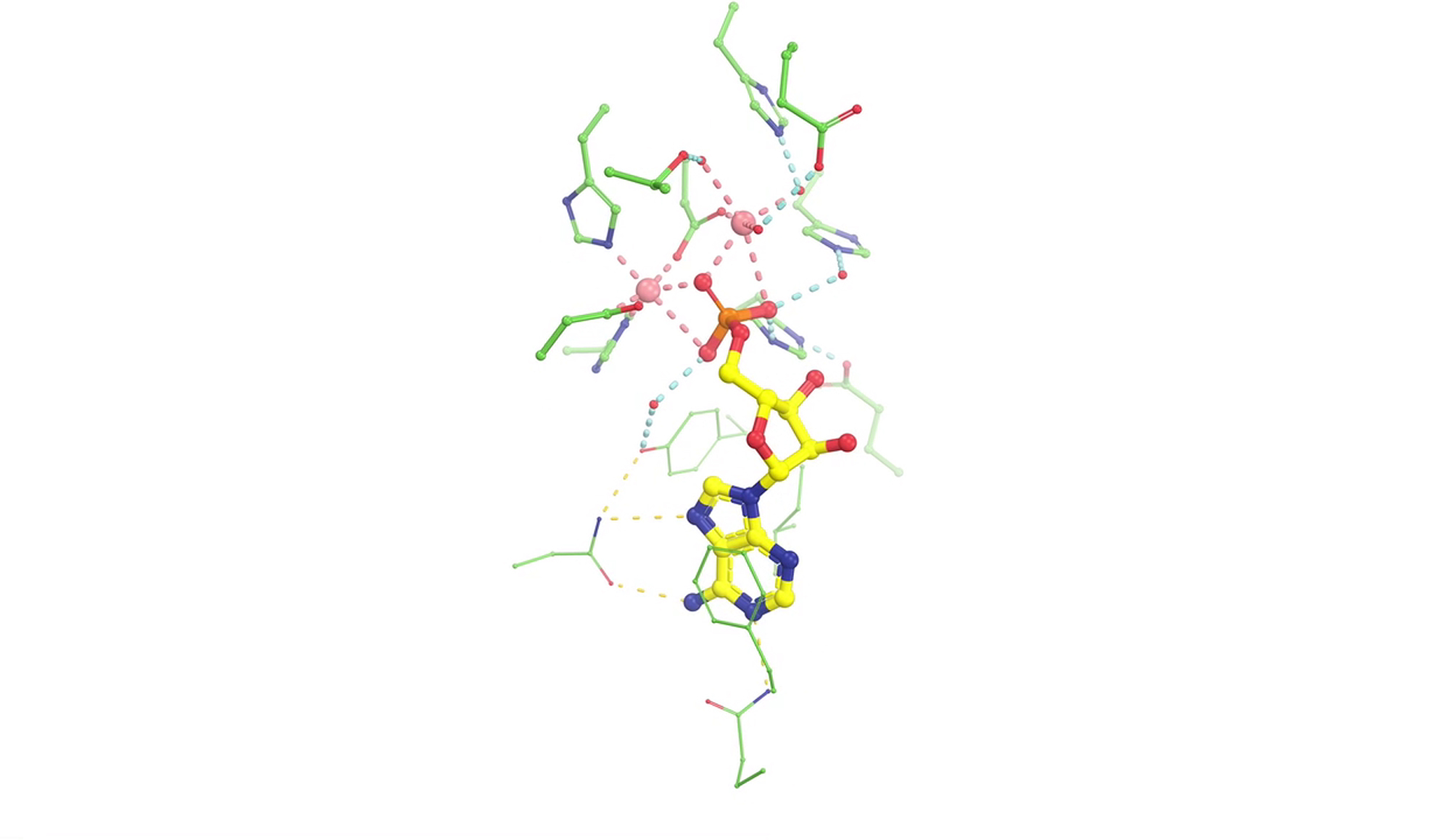

Supplement: mmcjpg [file NIHMS2039167-supplement-mmcjpg.jpg]
