# Supplementary material for: Chemical, Biochemical, and Structural Similarities and Differences of Dermatological cAMP Phosphodiesterase-IV Inhibitors
Source: J Invest Dermatol. Author manuscript; Available in PMC 2025 Jun 1. (PMC12103293; doi:10.1016/j.jid.2024.10.597)
Supplement: 1 [file NIHMS2039167-supplement-1.pdf]

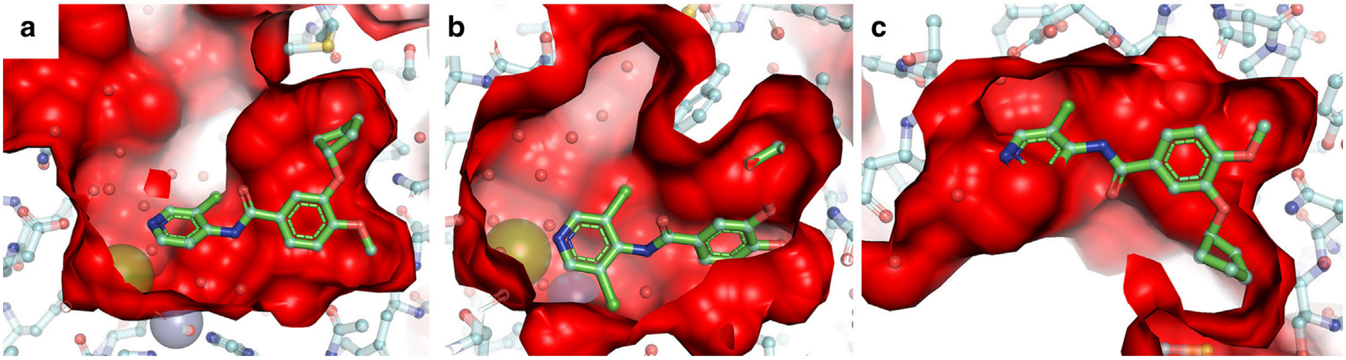

Supplementary Figure S1. Additional electrostatic potential surface representation of the inhibitor-binding pocket of the 1xm4/PDE4B−piclamilast complex structure in 3 different views a, b, and c, each emphasizing a slightly different orientation of the pocket. PDE4B, phosphodiesterase-IV B.
